# Modeling determinants of women’s decision-making autonomy on agriculture and household matters in rural Ethiopia: a mixed effects beta regression tree analysis

**DOI:** 10.1371/journal.pone.0355932

**Published:** 2026-09-10

**Authors:** Teshome Kabeta Dadi, Dessalegn Tamiru, Beyene Wondafrash, Mekdes Kebede Gebremariam

**Affiliations:** 1 Department of Epidemiology and Biostatistics, Faculty of Public Health, Institute of Health, Jimma University, Ethiopia; 2 Department of Nutrition and Dietetics, Faculty of Public Health, Institute of Health, Jimma University, Ethiopia; 3 Department of Community Medicine and Global Health, University of Oslo, Oslo, Norway; Innovative Aid, CANADA

## Abstract

Despite existing research on women’s decision-making autonomy (WDMA), there remains a significant gap in understanding the complex factors influencing women’s autonomy. This study investigated the determinants of WDMA in agriculture and household matters in rural Ethiopia. The study used data from nine rural districts in the Oromia and the former Southern Nations Nationalities and Peoples region in Ethiopia. A mixed effects beta regression tree model was used to identify variables impacting WDMA across diverse participant segments. The analysis incorporated various fixed effects, including family size, women’s and their partners’ years of education, women’s age, women’s perception towards cultural norms on gender differences, religion, household wealth quintiles, and participation in social groups. Random effects were taken into account by including the districts of the households and the survey rounds, while the gender of the household leader and marital status served as partitioning variables. Factors that were found to be significantly associated with WDMA among the married women from male-led households were: the age of the women, the perception of women towards cultural norms on gender difference, if women were Muslim compared to Orthodox Christian, richness of households and participation in social groups. In the case of women not in marriage and who were from male-led households, we identified the same factors to be associated with increased WDMA. Among women in female-led segments, only the age of the women and Orthodox Christianity compared to Muslim followers were contributing significantly to a higher WDMA. Interventions aimed at increasing women’s decision-making autonomy should consider acting on some of these determinants so that women can play a more prominent role in agricultural and household matters.

## Introduction

Decision-making autonomy refers to the ability to influence choices that impact various aspects of one’s life, in both personal and public domains [1]. Women’s decision-making autonomy (WDMA) in agriculture and households encompasses their capacity to influence or control decisions regarding farming practices, resource allocation, agricultural management, and household activities directly influencing household wellbeing [2]. It also includes their joint participation with men in visioning, planning,implementing, and monitoring activities, providing them with opportunities to make choices that affect their lives and that of their families’ [3–5].WDMA is often used as a proximate measure of women’s empowerment [6],and is believed to be essential for their overall development [7]. It is also believed to be crucial for boosting agricultural productivity, improving health and nutrition, and promoting gender equality which can allow women to control their lives and participate equally with men in family, community, and societal decision-making [8,9].Moreover, gender equality, WDMA, and overall empowerment of women are not only the means through which the fifth Sustainable Development Goal (SDG5) is achieved but also a foundational necessity for a way to achieve many of the targets of SDGs [10,11]. SDGs underlined global commitments to gender equality in agriculture in SDG2 (zero hunger) and SDG5(gender equality) goals [12]. The UN Food and Agricultural Organization (FAO) declared that sustainable agriculture and realizing the SDGs are unattainable without women’s empowerment [13].

In many developing countries, women play a vital role in agriculture and household activities, yet they often have limited decision-making autonomy [14,15]. Several studies have shown that solidification of WDMA can lead to improvements in their status, including greater control over resources, better health, and increased access to financial services, healthcare, skills development, income-earning opportunities, information about markets, and legal rights; which can positively impact agricultural productivity, nutrition, and food security [16–18].

Examining factors that impede or promote WDMA is thus essential, and several studies have explored factors affecting women’s decision-making autonomy. Studies across diverse regions, including Central Africa, India, and Ethiopia, indicate that women’s economic empowerment (WEE), financial access, and wealth status significantly influence their decision-making autonomy, especially in agriculture and healthcare [19,20].While financial constraints often limit women’s autonomy [21], greater wealth and community affluence tend to enhance WDMA [22–24],though one study from India reported women’s participation in household decisions decreased as family income increases [25]. Similarly, higher education, of both women and their spouses, consistently correlates with women’s improved participation in household and agricultural decisions, as shown in research from several Lower and Middle-income Countries (LMICs) [21,23,26–28].Studies in Ethiopia found that the higher education increased decision-making,concluded the found that higher education increased decision-making power, and improving education access strongly enhanced women’s autonomy [24,29–31]. Notably, educational parity between spouses and girls’ education was especially impactful in enhancing women’s authority and autonomy [7,32].

In addition to economic and educational factors, demographic characteristics such as age, family size [23,27,31], gender of household head [33], marital status [34–36], religion [24],and urban versus rural residence [37] were also found to influence women’s decision-making autonomy.Women’s employment status [23],asset ownership, and control over earnings, particularly through off-farm work, further empower their decision-making [38].Cultural norms and community perceptions play a pivotal role, with studies showing that gender norms often constrain women’s agency [23,27,29,30].However, positive attitudes towards women’s autonomy and involvement in social groups like Self Help Groups (SHGs) or cooperatives were found to significantly improve access to information and bolster decision-making power [21,39].

This study makes a significant contribution to the existing literature by being among the first in the Ethiopian context to model WDMA in agricultural and household matters using a mixed-effects BRT approach. Unlike previous studies that primarily relied on conventional statistical models, the proposed method effectively accommodates the bounded nature of the composite autonomy index, captures the hierarchical structure of the data, and identifies complex, non-linear relationships and interactions among predictors. Furthermore, the study extends the existing literature by incorporating previously underexplored determinants of WDMA, including women’s perceptions of gender-related cultural norms and household members’ participation in social groups, thereby providing a more comprehensive understanding of the factors associated with women’s decision-making autonomy in rural Ethiopia.

## Materials and methods

### Data source and study design

The study, titled “Evaluating Multisectoral Strategies for Enhanced Nutrition and Food Security in Ethiopia” also referred to as the Agriculture-Nutrition Household Panel Study,” was collaboratively conducted by Jimma and Hawassa Universities in Ethiopia and Tufts University in the USA. Anonymous data for the current study was accessed from Tufts University. The study collected data by interviews performed over two years in four rounds, encompassing 20 counties (kebeles) of 9 districts, 7 in Oromia and 2 in the former Southern Nations Nationalities and Peoples (SNNP) regions in Ethiopia. A sample of 1200 households was selected using multi-stage cluster sampling of woredas and kebeles.Households were sampled at the kebele level by the method of expanded program on immunization (EPI) [40,41].

## Measures and operational definitions

### Women’s decision making autonomy

The measurement of WDMA was done using eleven questions asked under the decision-making module of the female questionnaire of the Ag-Nutrition study [42]. The questions are reported in supplementary material S1 File in S1 Text. The internal consistency for the eleven items was assessed using Cronbach’s alpha. The alpha coefficient was 0.91, indicating an excellent reliability of the scale. The questions assessed the participation of women in decisions made about agriculture, access to income and expenses in their respective households, and freedom of using family planning by the women. In this study, autonomy refers to a woman’s having the final say, either independently or jointly with her husband/partner, on household and agricultural decisions. Each question allowed for 8 answers. Re-coding was done to the responses to a three-response variables (2= for women who were able to decide individually, 1= for women who were able to decide jointly with their husbands, and 0 = otherwise), in the same way as previously reported elsewhere [43–45]. That was because the 11 questions from which women’s decision-making autonomy scores were calculated were not asked to all the households included in the study since the questions were not relevant in some households because decisions were not made and re-normalization was needed. Therefore, we used the mean score of WDMA,ms_wdma, which is the total score of WDMA score_wdma divided by the number of questions asked to the same woman as shown in Eq (1).


$$\begin{array}{c} ms\_wdma=\frac{score\_wdma}{\text{Number of questions actually answered by the woman}} \end{array}$$ (1)

### Social participation

Social participation was assessed by asking whether any member of the households participated in any of the eight social groups in their locality. The social groups include a farmers’ group, a women’s group, a religious group, a youth group, a health-related/well-being group, a financial-related group, a local administration group, and other types of groups if any. The responses for each question were dichotomous, with 0 for no participation and 1 for participation in each of the group types. The overall participation in the social group was then assessed as no participation coded as ‘0’, if none of the members of the household participated, and defined as ‘has participation’ coded as ‘1’, if either of the household members participated in at least one of the group.

### Women’s perception towards cultural norms on gender difference

Women’s perception towards cultural norms on gender difference was assessed based on 5 statements which had 4-point Likert scale responses: 1 = strongly agree, 2 = agree, 3 = disagree, 4 = strongly disagree. The statements were that ‘Women cannot stand up for their own choices’, that ‘the female child is the internalization of the low value by society’, ‘Female children are socialized to eat less’, ‘Female children are socialized to eat last’, and ‘Female children are socialized to eat leftovers’. The participants were asked these questions and they responded with their levels of agreement or disagreement with the five statements. The scores of the five questions with 4 responses were computed for all the participants, which varies from 4 to 20 with mean and median values of 15.8 and 15.0 respectively.

### Household wealth Quintiles

This study was conducted in rural districts of Ethiopia and thus the lists of items used in the determination of household wealth quintiles were the lists of productive assets owned by the households considered through the study periods. First, a composite wealth index was determined at household level using principal component analysis. The items were 11 in number and their responses were coded as ‘0’ if the households didn’t own the item and coded as ‘1’ if they owned the productive assets. Then, the households were grouped into five wealth quintiles [46]. See the supplementary file online for details S2 File in S1 Text.

### Survey rounds

The study was repeated four times over a period of two years. Thus, we expected that a learning effect might exist as women could have had further thoughts about the question they answered in the first round, providing a slightly different answer during the second survey round. Thereafter, the learning effect might be over. To address this, we created a covariate that combined the second, third, and fourth rounds into a ‘Not Baseline’ category, while designating the first round as ‘Baseline’, and incorporated this dichotomous survey round variable as a random effect in all models to account for individual learning effects that are assumed to be similar across all participants. Additionally, we evaluated the interclass correlation to check for a clustering effect of the survey rounds on the outcome variable WDMA, finding no significant clustering across all four rounds. Clustering effect between the ‘Baseline’ (first round) and ‘Not Baseline’ (merged last three rounds) groups was also very small, with an ICC value of 0.0053, indicating minimal between-group variance and suggesting that any clustering effect due to survey rounds is negligible. This random effect was however retained in the models to control for potential residual effects.

### Statistical analysis

The outcome variable of the current study was WDMA, which was calculated by taking the score of the 11 questions asked for the main woman about decisions made in the household. The average score was then calculated as per the number of decisions made in the household to get the mean WDMA as described above. The mean varied between 0 and 2 inclusive of the two limits, i.e.,[0, 2]. Then, two transformations were made on this mean score. One was to bring the values to be in the range of [0, 1] by Eq (2) and the second one was the transformation of relaxing the two extremes, 0 and 1, Eq (3) so that its range is in (0, 1) for the purpose of fitting Beta regression model.


$$\begin{array}{c} norm\_wdma=\frac{ms\_wdma-0}{2-0}=\frac{ms\_wdma}{2} \end{array}$$ (2)

$$\begin{array}{c} trans\_wdma=\frac{norm\_wdma\cdot (n-1)+0.5}{n} \end{array}$$ (3)

The beta regression model is a statistical technique used for modeling continuous variables that assume values in the open standard unit interval, (0, 1) [47,48]

The beta distribution is a continuous probability distribution defined over the unit interval with density function,


$$\begin{array}{c} \pi (y,p,q)=\frac{\Gamma (p+q)}{\Gamma (p)\Gamma (q)}y^{p-1}(1-y{)}^{q-1},\;0<y<1 \end{array}$$ (4)

where *p*, *q* > 0 are indexes, ϕ=p+q, and Γ(.) is the gamma function in such a way that


$$\begin{array}{c} E(y)=\frac{p}{p+q}=\mu \end{array}$$ (5)

and


$$\begin{array}{c} var(y)=\frac{pq}{\left(p+q{)}^{2}(p+q+1\right)}=\frac{\mu (1-\mu )}{1+\phi }=\frac{V(\mu )}{1+\phi } \end{array}$$ (6)

are the mean and variance of y, respectively. Moreover, ϕ is a precision parameter interpreted as: for a fixed μ, an increase in the value of ϕ results in a decrease in the variance of y. Its model in terms of the mean and precision is then.


$$\begin{array}{c} \;f(y;p,q)=\frac{\Gamma (\phi )}{\Gamma (\mu \phi )\Gamma ((1-\mu )\phi )}y^{\mu \phi -1}(1-y{)}^{(1-\mu )\phi -1},\;0<y<1, \end{array}$$ (7)

where 0<μ<1 and ϕ>0. The shapes of the distribution is dependent on the mean and precision parameters. The corresponding regression model for this beta distributed data was implemented by the betareg package in R which was based on the classical Maximum Likelihood (ML) inference [47,49]. In this analysis, logit link function is used to transform the mean response and hence the regression parameters can be interpreted in terms of change in relative proportion which is similar to the odds ratio.

Previous works have reported that although the *betareg* package is comprehensive, it practically requires additional techniques since the ML inference can be biased, leading to underestimated standard errors and also capturing all data heterogeneity through linear predictors is challenging, especially with latent groups/clusters [50]. To enhance the practical application of beta regression to provide more reliable inference and capture both observed and unobserved/latent heterogeneity in the data, beta regression is extended in three ways: bias correction (BC) or bias reduction (BR) of the ML estimator, beta regression tree models by means of recursive partitioning and latent class beta regression through finite mixture models were suggested [51]. The potentially biased inference corrections designed was extended to the case with two parameters and the methods are embedded in *betareg()* in recent versions of the betareg so that the software allows for BC or BR estimation by adopting the unifying iteration developed by Kosmidis and Firth [50]. Model-based recursive partitioning (MOB) [52] and finite mixture models [53,54] are applied to beta regression to address the presence of heterogeneity problem between groups/clusters of observations. The concept behind both methods is to identify situations where the regression relationships differ among various groups within the population. MOB is suggested to avoid model complexity.It also facilitates interpretability and solves the unnecessary complexity introduced if the differences are only present in a subset of the combined groups induced by several variables that are directly included in the regression relationships. The function *betatree()* provides model-based recursive partitioning of beta regressions leveraging tools from the *partykit* and *betareg* packages [55]. On the other hand, if groups cannot be directly related to observed variables, the heterogeneity can be accounted for by using finite mixture models through the *betamix()* function provides beta regression mixture models (or latent class beta regression) reusing the generic functionality from the flexmix package [56].

In this study, the inferences obtained from the BC and BR models were identical to those resulting from the ML model inferences, leading us to forgo further exploration of the BC or BR models. (See the third supplementary material S3 File in S1 Text). The second extension of the beta regression, known as beta regression trees (model-based recursive partitioning), provided the best fit to the data. In this study,most inferences were therefore based on this model, as it effectively captured heterogeneity in the outcome by estimating separate regression coefficients for each homogeneous subgroup. On the other hand, gender of the head of the household and marital status of the main women in the household were used as splitting variables to partition all the study subjects into different subgroups, representing segments of the tree with the goal of creating branches that are homogeneous in terms of the regression associations with the response variable. Parameter estimation was done internally in each segment once the requirement for parameter stability test was checked: stability was investigated by structural change test statistics together with the corresponding p-values obtained using the *sctest()* function in R [51].

In the current study, a mixed-effects beta regression tree model was employed, with districts (9 woredas) and survey rounds (2 rounds) as random effects to manage variability across clusters, woreda and survey rounds. Chena district and baseline survey round served as the reference woreda and survey rounds respectively.

We fit various types of beta regressions and finally the best model of all, as assessed by Akaike Information Criterion (AIC), was used to infer the parameters of interest. To assess whether the association between WDMA and covariates is different for some segments in the data, MOB methods through Beta Regression Tree (BRT) was used with the aim of capturing differences in distribution that are not yet adequately described by the explanatory variables. Then, based on the variable dispersion beta regression, we assess whether the parameters of this variable dispersion beta regression are unstable across the intended partitioning variables.

Model comparisons of the classical beta regressions with and without interactions, and with and without random effects with that of the beta regression tree was done by the AIC. Based on this criteria, we found that the beta regression tree was the best of all the models under comparisons.

We also refitted a beta regression for each segment of a beta tree by ML, BR, and BC methods. Again, an analysis of the marginal effects of the covariates on the mean score of WDMA was also done for better interpretability. Marginal effects are the partial derivatives (dy/dx, where dy and dx are the derivative of the outcome variable and the derivatives of each explanatory variable) of the regression equation for each variable in the model for each unit of data. Average marginal effects (AMEs) are the average of these unit-specific partial derivatives over a sample [57]. The margins in R margins() method was used to obtain AMEs and its summary() to make the estimates of the mean model of Beta regression more interpretable. These analyses were done after refitting the mixed effects beta regression model for each subgroup of the data partitioned by the BRT. The necessary model selection was done among ML, BC, and BR models by the AIC method. The BR model was the best in all three segments and hence the inference and the calculation of AMEs was based on these selected models. Refer to supplementary materials S4 File in S1 Text.

Refer to the diagnostic plots for the first segment of the data formed by BRTM in supplementary material S1 Fig in S1 Text.

### Summary of model assumptions

In summary, a Mixed Effects Beta Regression Tree Model assumes that the response variable is continuous and restricted to the open interval (0,1), following a Beta distribution with typically the logit link relating the mean response to the predictors. The model also assumes that the data have a hierarchical or clustered structure, with random effects accounting for within-cluster correlation, while clusters are independent and random effects are approximately normally distributed. Unlike traditional beta regression, the tree component does not require linear relationships or pre-specified interaction terms, as it automatically captures complex nonlinear effects and high-order interactions through recursive partitioning. To ensure reliable inference, the model further assumes adequate observations within terminal nodes and appropriate tree complexity [47,52].

### Ethical considerations

The protocol for this study was approved by Social, Behavioral, and EducationalmResearch (SBER) Institutional Review Board of Tufts University in USA and Jimma University Institutional Review Board in Ethiopia. We did secondary data analysis for the current study following requesting access to data from Tufts University. Moreover, none of the authors had access to any personally identifiable information about participants at any point of the study, ensuring confidentiality throughout the study and compliance with ethical standards.

## Results

### Study characteristics

The mean age of the women included in the four rounds of the study was 36.2 (sd 11.4). The average score of WDMA was 1.00 with a standard deviation of 0.43 and the range was from no participation in decision-making (mean score of 0) to sole decision-making by women (mean score of 2). Islamic religion followers constituted the majority of the study participants (66.5%) followed by Orthodox religion followers (21.7%). Most of the households (89%) were led by men. Eighty-nine percent of the women participating in the study were married, only 8% were single, and the rest of the women were divorced or widowed. For more details on the study participants, refer to Table 1.

**Table 1:** Characteristics of women who participated in the agriculture and nutrition household panel study.

Characteristics	n = 4,703
Mean score of wdma	0.00, 1.00(0.43), 1.00, 2.00 (0.86, 1.10)^*1*^
Partner’s years of education	0, 2(3), 0, 15 (0, 4)^*1*^
Women’s years of education	0, 1(3), 0, 13 (0, 2)^*1*^
Family Size	1, 6(2), 5, 13 (4, 7)^*1*^
Women’s age	17, 36(11), 35, 80 (27, 45)^*1*^
Score of perception towards gender difference	4, 16(3), 15, 20 (14, 19)^*1*^
**Categorical Variables**	**Frequency (%)**
**Gender of household leader**	
Male Lead	4,156 (89%)
Female Lead	510 (11%)
**Household Wealth Quintile**	
Very Poor	961 (20%)
Poor	931 (20%)
Medium	936 (20%)
Rich	936 (20%)
Very Rich	939 (20%)
**Participation in Social Group**	
No Participation	624 (13%)
Has Participation	4,079 (87%)
**Study round**	
First round	1,192 (25%)
Second round	1,182 (25%)
Third round	1,173 (25%)
Fourth round	1,156 (25%)
**Baseline or not**	
Baseline	1,192 (25%)
Not Baseline	3,511 (75%)
**Women’s religion**	
Muslim	3,070 (66%)
Orthodox	1,000 (22%)
Protestant/Catholic	547 (12%)
**Marital status**	
Married	4,064 (89%)
Never Married	374 (8.2%)
Divorsed/Widowed	125 (2.7%)

^*1*^Minimum, Mean(SD), Median, Maximum, (25, 75 percentiles)

The range of values of the transformed WDMA is in a bounded interval between 0 and 1, which can be interpreted as a percentage. A 0% indicates a complete lack of autonomy in decision making and 100% is fully autonomous by the woman in making decisions and hence suitable modeling by the beta regression model. This standardized mean score of WDMA has an asymmetric distribution and so the median is a more appropriate measure of central tendency. However, the standardized median score is nearly equal to the standardized mean score, which is 0.5.

### Results from mixed effects beta regression tree model

The BRT algorithm partitioned the study subjects into three homogeneous segments by iterative parameter stability tests. Three outer nodes at which the stability requirement is satisfied were identified in the current study, which represents the three homogeneous segments of the study participants with respect to WDMA. The data was split into three subgroups with stable parameters estimated for each segment. The first segment/node 3 contains 3961 households led by male with married women; the second segment/node 4 contained 123 male-led households but with single, divorced, or widowed women; and the last segment/node 5 contained 408 female-led households. Refer to the supplementary material S2 Fig in S1 Text to visualize the beta regression tree plot.

### Results for households led by males with married women (first subgroup)

As described above, this subgroup constitutes 88.2% of the households. We found the following covariates to be statistically significantly associated with WDMA: age of the women (β=.0034,p-value/*p* = .0237), perception of women towards cultural norms on gender difference (β=.0391,*p* = .0000), being a follower of Orthodox Christianity compared to Muslim followers(β=−.1867,*p* = .0000), being from the richest households compared to the poorest (β=−.1540,*p* = .0011), and participation in social groups compared to non-participation (β=.1180,*p* = .0052).

In addition to the mean model, beta regression incorporates the precision model which captures the variability of the dependent variable through precision estimates. The negative sign of the significant precision parameter shows a decrease in precision which means an increase in the overall variance of the dependent variable whereas, a positive sign indicates an increase in precision, a decrease in the overall variance of the dependent variable keeping other variables constant. With this respect, Gera, Goma, and Limu Seka districts were associated with an increase in precision while Enamor Ener, Gasara, Gechi, and Wonchi districts were all associated with decreases in precision compared to Chena district keeping other variables constant. However, no significant difference in precision was observed between Dedesa and Chena districts. Similarly, there was a statistically significant difference in precision observed between the non-baseline survey round and the baseline one. Non-baseline survey round is associated with an increase in precision, assuming other predictors are constant.

### Results for households led by males with women not in marriage (second subgroup)

The second segment contained 2.7% of the total households,made up of male-led households and women who had never married, widowed, or divorced. The age of the women was significantly associated with WDMA for this subgroup but in the opposite direction of the first subgroup (β=−.0112,*p* = .0107). The perception of women towards cultural norms on gender difference was also significant for this subgroup (β=.1173, *p* = .0008). Another variable found to have a significant association with WDMA was the household wealth quintiles. The coefficients of being from the poor, middle, rich, and richest households compared to the poorest were (β=−1.5786,*p* = .0000), (β=−1.0277,*p* = .0027), (β=−1.3538, *p* = .0001), (β=−1.0596,*p* = .0012) respectively.

In this subgroup, unlike in the first segment, Dedesa woreda had a significant precision estimate next to Limu Seka woreda. Limu Seka and Dedesa woredas were associated with an increase in precision or decrease in the overall variance of the dependent variable with precision coefficients of (β=−5.1598, *p* = .0000), (β=.9710, *p* = .0020) respectively at constant values of other predictors.

### Results for female-led households (third subgroup)

The model included 9.1% households that were led by women. For this segment of the data, only two variables showed a significant association with WDMA: the age of the women and being a follower of Orthodox Christianity compared to Muslim followers (β=−.0136 with *p* = .0183) and (β=.3916 with *p* = .0170) respectively. None of the precision coefficients was found significant in this subgroup. For further details of the mixed effects beta regression tree results across each segment, refer to Table 2

**Table 2:** Results from Beta Regression Tree on determinants of women’s decision making autonomy on agriculture and household matters using data from agriculture and nutrition household panel study.

		Node 3^*1*^ (n = 3961)		Node 4^*2*^ (n = 123)		Node 5^*3*^ (n = 408)	
	**Covariates**	**Estimates(SE)**	**P-Value**	**Estimates(SE)**	**P-Value**	**Estimates(SE)**	**P-Value**
	(Intercept)	−0.8312(0.1029)	6.5e-16 *** ^*5*^	−0.5450 (0.6115)	0.3729	1.7434(0.4852)	0.0003***
	Family Size	−0.0138(0.0075)	0.0649.	0.1162(0.0598)	0.0521.	0.0005(0.0389)	0.9892
	W-Education Years^*4*^	0.0124(0.0068)	0.0661.	−0.0302(0.0292)	0.3018	0.0013(0.0192)	0.9475
	Age of women	0.0034(0.0015)	0.0237*	−0.0112(0.0050)	0.0107*	−0.0136(0.0058)	0.0183*
	Cultural Norms	0.0391(0.0044)	<2e-16***	0.1173(0.0349)	0.0008***	−0.0166(0.0198)	0.4014
	**Religion of Women**						
	Muslim1						
	Orthodox2	−0.1867(0.0373)	5.4e-07****	−0.2620(0.2712)	0.3339	0.3916(0.1640)	0.0170*
	Protestant/Catholic3	0.0096(0.0456)	0.8332	0.1227(0.3099)	0.6921	0.2593(0.2462)	0.2922
	**Wealth Quin-tiles**						
	Poorest1						
	Poor2	0.0469(0.0467)	0.3154	−1.5786(0.3617)	1.3e-05***	0.1608(0.1605)	0.3164
	Middle3	−0.0028(0.0468)	0.9529	−1.0277(0.3425)	0.0027**	−0.0973(0.1995)	0.6259
	Rich4	−0.0161(0.0458)	0.7254	−1.3538(0.3537)	0.0001****	−0.2791(0.2310)	0.2269
	Richest5	−0.1540(0.0472)	0.0011**	−1.0596(0.3273)	0.0012**	0.0037(0.2183)	0.9866
	**Social Participation**						
	No0						
	Yes1	0.1180(0.0423)	0.0052**	−0.3216(0.1818)	0.0769.	0.2389(0.1718)	0.1644
	(Intercept)	1.0731(0.0713)	<2e-16***	−0.1504(0.2821)	0.5941	−0.3858(0.2399)	0.1078
	**Districts**						
	Chena1						
	Dedesa2	−0.1472(0.0868)	0.0898.	0.9710(0.3145)	0.0020**	−0.4520(0.2622)	0.0848.
	Enemor Ener3	−0.3431(0.0913)	0.0002***	−0.3485(0.3636)	0.3378	−0.0064(0.2492)	0.9794
	Gasara4	−1.0265(0.0834)	<2e-16***	−1.2695(1.0536)	0.2282	−0.1577(0.2713)	0.5609
	Gechi5	−0.5020(0.0981)	3.1e-07***	−0.2012(0.4034)	0.6179	−0.4327(0.2713)	0.1363
Precision Model	Gera6	0.2825(0.0976)	0.0038**	−0.0881(0.4938)	0.8585	−0.2547(0.2959)	0.3895
	Goma7	0.4563(0.0916)	6.3e-07***	0.4320(0.3947)	0.2737	−0.2493(0.2820)	0.3766
	Limu Seka8	0.2645(0.0764)	0.0005***	5.1598(0.6209)	<2e-16 ***	−0.1798(0.2670)	00.5005
	Wonchi9	−0.2499(0.0855)	0.0035**	−0.1663(0.3393)	0.4148	0.0161(0.2973)	0.9567
	**Survey rounds**						
	Baseline0						
	Not Baseline1	0.4330(0.0450)	<2e-16 ***	−0.2460(0.2148)	0.2522	0.1779(0.1145)	0.1204
	**Log-likelihood(Df)** ^***6***^	**678.9(22)**		**113.5(22)**		**1172 (22)**	
	**Number of iterations**	**41(BFGS)+3(Fisherscoring)**		**39(BFGS)+4(Fisherscoring)**		**45(BFGS)+ 3(Fisherscoring)**	

^*1*^ Male-led households with married women, ^*2*^ Male-led households with never married/widowed/divorced women, ^*3*^ Female-led households

^*4*^ W-Education = Women’s Education, ^*5*^ Signif. codes: ‘***’ 0.001 ‘**’ 0.01 ‘*’ 0.05 ‘.’ 0.1 ‘ ‘ 1; ^*6*^ Df = Degree of freedom

### Average marginal effects of covariates on WDMA

Regardless of the differences in the values of the estimated coefficients, the covariates found significant in both the BRT and Beta regression model for the first and the third subgroups separately were identical but in the latter type of model, the age of the women and the perception of the woman towards cultural norms on gender difference were found insignificant unlike for the second segment (only the categories of wealth quintiles were significant). It can be concluded that the BRT algorithm is more powerful than the ordinary Beta regression model in detecting the small difference with a relatively smaller sample size than the ordinary Beta regression model fitted separately.

**Fig 1:**
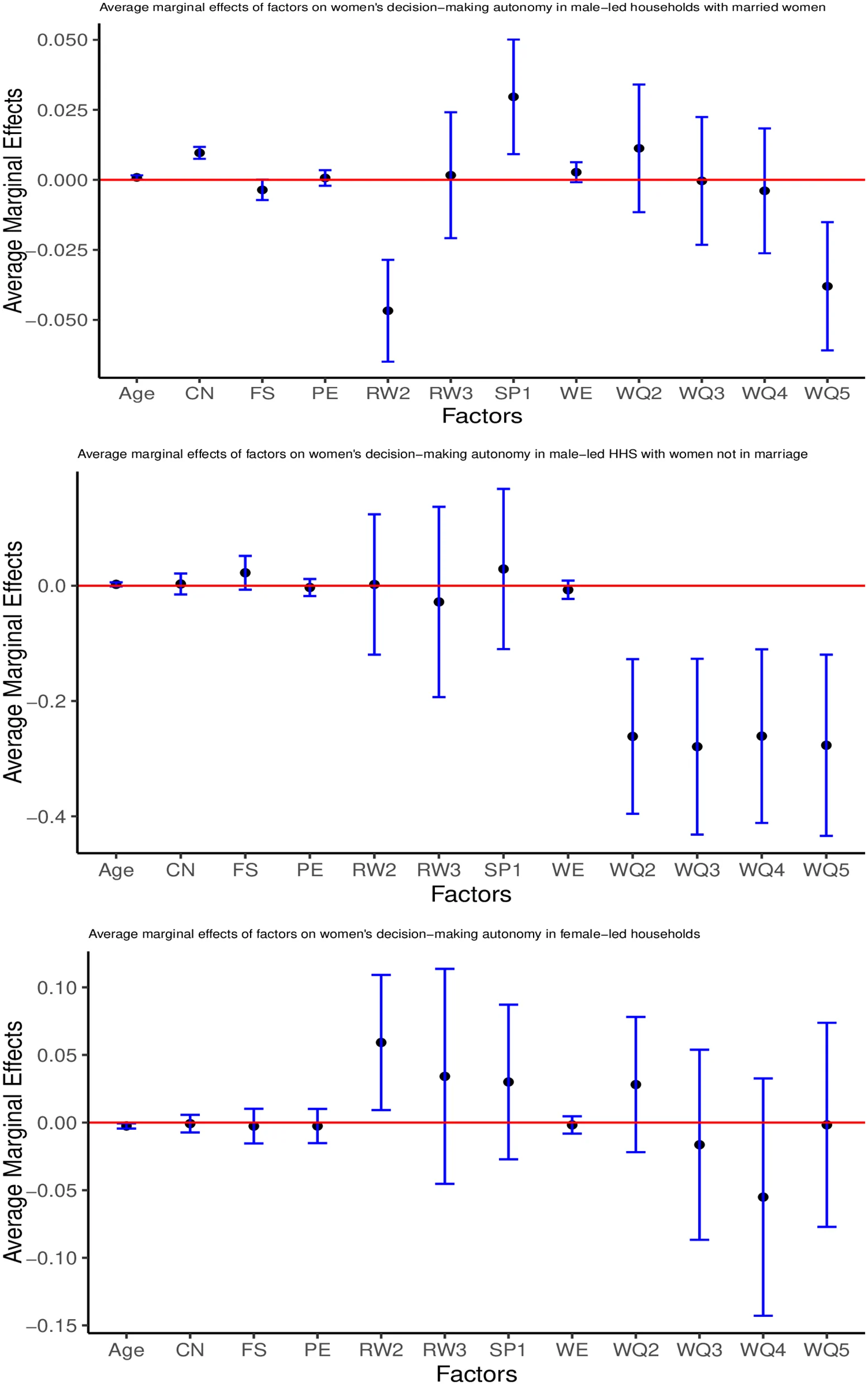
Average marginal effects of co-variates on WDMA across the three segments from the refitted beta regression segmented by BRTM. We abbreviated the names of covariates included in the plots of marginal models as follows: Family Size (FS), Women’s Education Years (WE), Partner’s Education Years (PE), Age of women (Age), Cultural Norms (CN), Religion of Women (RW: 1-Muslim, 2-Orthodox, 3-Catholic/Protestant), Wealth Quintiles (WQ: 1-Poorest, 2-Poor, 3-Middle, 4-Rich,5-Richest), Social Participation (SP: 0-Not Participated, 1-Participated).

For married women from male-led and for women from female-led households, the proportion of WDMA increased by 0.0009 and decreased by 0.0025 respectively when the age of the women increased by one year, keeping other values constant. Age has no marginal effect on WDMA among male-led households with single, divorced, and widowed women. The religion of the women had a significant effect on WDMA for the first and third segments of the households but its effect was opposite in both segments. The proportion of WDMA decreased by 0.0468 for Orthodox Christianity followers compared to Muslim women for married women from male-led households, while it increased by 0.0592 in the third segment(female-led households), keeping other values of the predictors constant. Wealth status was one of the variables found significant in the first and second segments, but not in the third segment. The proportion of WDMA decreased by 0.0380 as the household wealth quintile changed from the richest households to the poorest households for married women from the male-led households. Similarly, other categories of wealth status compared to the poorest household wealth quintiles had a decreased effect on the proportion of WDMA in the second segment(male-led households of women not in marriage). Accordingly, the proportion of WDMA among the poor, middle, rich, and richest households decreased by 0.2614, 0.2792, 0.2609, and 0.2768 respectively compared to the poorest wealth quintile. Finally, the perception of women towards cultural norms on gender difference (perception of women on gender equality), and participation of women in local groups were significant only for married women who were from male-led households. Meaning, as the score of the gender equality measure increased by one score, the proportion of WDMA increased by 0.0096 with fixed values of other predictors. Regarding participation in the local groups, the proportion of WDMA increased by 0.0296 for those who participated in social groups compared to those who had no participation. The details about marginal effects of each covariate on WDMA were summarized in Table 3 and the corresponding marginal effect plots across the three segments was also depicted in Fig 1.

**Table 3:** Marginal effects of factors on women’s decision-making autonomy on agriculture and household matters using data from agriculture and nutrition household panel study.

Factors	Node 3 (n = 3961)		Node 4 (n = 123)		Node 5 (n = 408)	
	Estimates(SE)	(95% CI)	Estimates(SE)	(95% CI)	Estimates(SE)	(95% CI)
Family Size	−.0036(.0019)	(−.0072,.0000)	.0225(.0149)	(−.0068,.0517)	−.0026(.0065)	(−.0154,.0102)
W-Education Years^*1*^	.0027(.0018)	(−.0008,.0063)	−.0070(.0081)	(−.0230,.0089)	−.0017(.0033)	(−.0082,.0047)
P-Education Years ^*2*^	.0007(.0014)	(−.0021,.0035)	−.0031(.0075)	(−.0178,.0117)	−.0026(.0065)	(−.0152,.0101)
Age of women	.0009(.0004)	(.0001,.0016)*^3^	.0024(.0018)	(−.0011,.0059)	−.0025(.0010)	(−.0045,-.0006)*
Cultural Norms	.0096(.0011)	(.0075,.0118)*	.0030(.0092)	(−.0151,.0211)	−.0008(.0033)	(−.0073,.0057)
**Religion of Women**						
Orthodox1	−.0468(.0093)	(−.0649,-.0286)*	.0021(.0621)	(−.1195,.1238)	.0592(.0255)	(.0092,.1092)*
Protestant/Catholic2	.0016(.0115)	(−.0209,.0241)	−.0281(.0842)	(−.1932,.1370)	.0342(.0406)	(−.0454,.1137)
**Wealth Quintiles**						
Poo2	.0113(.0116)	(−.0115,.0340)	−.2614(.0684)	(−.3955,-.1273)*	.0281(.0255)	(−.0219,.0781)
Middle3	−.0004(.0116)	(−.0232,.0224)	−.2792(.0778)	(−.4316,-.1267)*	−.0164(.0359)	(−.0867,.0539)
Rich4	−.0039(.0114)	(−.0262,.0184)	−.2609(.0768)	(−.4115,-.1103)*	−.0551(.0448)	(−.1429,.0327)
Richest5	−.0380(.0117)	(−.0609,-.0151)*	−0.2768(0.0802)	(−.4339,-.1196)*	−.0017(.0385)	(−.0771,.0738)
**Social Participation**						
Yes1	.0296(.0104)	(.0092,.0501)*	.0290(.0709)	(−.1099,.1679)	0.0300(.0292)	(−.0272,.0872)

^*1*^ W-Education = Women’s Education, ^*2*^ P-Education = Partners’ Education, ^*3*^ Significant at 95% CI

Estimate=dy/dx, SE=Standard Error of dy/dx

Node 3 = Male-led households with married women

Node 4=Male-led households with never married/widowed/divorced women

Node 5 = Female-led households

## Discussion

### Comparing beta regression tree model & separate beta regression models

We used the Mixed Effects Beta Regression Tree to model the determinants of WDMA about agriculture-related and household matters using household panel study from rural Ethiopia. The Beta Regression Tree was extended from the Beta Regression model by incorporating the ability to use a decision tree structure, which allows the model to split the data into different segments and fit a different Beta Regression model to each segment by MOB method. This enables the model to capture more complex relationships between the variables, as it fits different curves to different segments of the data and this makes the model more powerful and versatile than the Beta Regression model without tree [51].

Regardless of the values of the mean and precision coefficients, the covariates found significant from both types of modeling were similar except for the difference in the second segment. The second segment contains data from 123 male-led households with women either single, divorced, or widowed. As compared to the separate beta regression, perception of cultural norms towards gender difference and family size were additional covariates found significant in BRTM but only the categories of wealth quin-tiles were significant compared to the poorest in the separate beta regression models implying that the BRTM is better than the ordinary Beta regression in capturing the complex associations even in small segment sizes.

### Households led by males with married women

The link used in fitting the model determines the interpretation of the estimated parameters. When the logit link function is used to transform the mean response, the regression parameters can be interpreted in terms of the odds ratio (OR) or relative proportion in the context of Beta regression [47]. From now onwards, interpretations for all significant covariates resulting from the BRTM were done in the sense of relative proportions.

The first segment of the data contains male-lead households with married women. For this segment, the age of the women, the perception of the women towards cultural norms on gender difference, religion(Orthodox vs. Muslim), wealth quin-tiles(richest vs. poorest), and participation in social groups (yes vs no) were significantly associated with WDMA. An increase in the age of married women from the male-led households was positively associated with WDMA. A year increase in the age of the women increased the relative proportion of WDMA by 0.34%. This finding is aligned with findings of other studies conducted in different African countries including Ethiopia as well as Asia, that reported older age was positively related to decision-making autonomy [25,26,34,58]. Other studies from Ethiopia have also documented that age has a significant impact on women’s agricultural participation [35], and positively impacted women’s empowerment in general [59].

Our study also uncovered the importance of a determinant that was less studied quantitatively, namely the perception of women towards cultural norms on gender differences. Positive perception towards gender equality by the women had a positive effect on WDMA. As the score of perception of women towards cultural norms on gender differences increased by one score, the relative proportion of WDMA increased by 4%, which is a quite small impact. This result is in line with studies conducted in Indonesia that reported men and women have different perceptions about women’s decision-making in agriculture in which women’s roles in at least joint decisions are smaller due to the influence of social norms on spouses’ perceptions of decision-making participation [27]. Another study from Myanmar also uncovered the contextual norms that women are often seen as farm ‘helpers’ rather than farmers, with these gender norms restricting women’s access to technical learning opportunities and their roles in agriculture and decision-making [60].Previous studies in Ethiopia also revealed that social or customary norms hinder women’s agency, women’s effective land use and participation in decisions, and their ability to innovate [36,61],while favorable attitudes improve women’s autonomy [62]. Religion was found as one of the significant determinants of WDMA among male-led households with married women. At fixed values of other predictors, Muslim religious followers had an increased relative proportion of WDMA by 17.03% compared to the Orthodox religious followers. This finding is against a comparison study about Indian and Pakistani women’s autonomy which reported that Muslim women exert about as much autonomy in their lives as Hindu women [63]. Similarly, the World Values Survey also stated religiosity was strongly correlated with gender inequitable attitudes across countries though it was impossible to identify a single religion that stands out as more gender inequitable than others [64]. This finding appears to go against the common opinion that women from Orthodox Christianity seem to be more autonomous than Muslim women. A possible reason for our finding could be that the Orthodox Christianity followers were a minority in the Muslim-majority areas of our study, and possibly this belonging to a minority group might encourage more traditional gender norms that restrict women’s participation in decision-making. Instead, Muslim women as a majority may have relaxed practices that allow for better participation in decision-making, especially on decisions related to household aspects. The impact of religion on the participation in decision-making of women in rural African communities deserves further investigation.

Married women who were from the male-led and richest household wealth quintile had a decreased relative proportion of WDMA by 14.27% compared to those from the poorest wealth quintile households of married women who were from the male-led households. This finding is not aligned with the common expectation that being a member of the wealthiest household is a guarantee to participate more in decision-making on agriculture and other household aspects. This does not appear to be the case for Ethiopian married women and richest male-led households. These results align with a study from India that reported women from the richest and rich households were less likely to participate in decision-making compared to women from the poorest households [58]. Another study from Uganda also reported that wealthier households exhibit more male-dominated decision-making patterns, while women’s bargaining power is diminishing as the household wealth status improves, and joint decision-making occurs more in poorer households [65]. On the other hand, a study in Ethiopia reported that higher autonomy regarding maternal and child health care was linked to higher household income [62]. This difference could be related to the area of decision-making which differs between this latter study and our study.

Participation in social, economic, cultural other groups by any of the household members is anticipated to enhance the WDMA in their households. The current findings confirm this, as women from the households who participated in local groups had increased WDMA, by a relative proportion of 12.5% compared to women from non-participating household members. The current finding is consistent with the result from a study in India which reported that participation in SHGs encourages joint decision-making in agriculture, and that women’s participation in SHGs increased decision-making roles by 8–13% [66]. According to other studies in India and Vanuatu, participation of women in community activities or SHG increases women’s access to information and enhanced their confidence for better participation in decision-making of the households [21,67,68]. Similarly, studies from Uganda, showed that membership in different co-operatives and farmers’ groups enhances women’s autonomy and positively impacts women’s economic well-being and decision-making [32,39].

### Households led by males with women not in marriage

This segment contains male-led households with women who were either single, divorced, or widowed. For this sub-group, there was a statistically significant association between the age of the woman, the perception of women towards cultural norms on gender difference, and wealth (all the top four levels of the household wealth quintiles compared to the bottom) with WDMA.

Unlike in the first segment, an increase in the age of women had a decremental effect on the WDMA for women not in marriage from male-led households. A year increase in the age of women decreased the relative proportion of WDMA by 1.11%. There can be several reasons why the higher the age for women, who were either single, divorced, or widowed, and from male-led households, the lower the decision-making power in their households. First, women who are single, divorced, or widowed but live in male-headed households may have decision-making roles diminishing as their age increases due to loss of rights other than staying in the households, especially for those women who live as dependents. Secondly, if the women are widowed or divorced, they might possibly transfer their decision-making power to their male children or to some other male to make decisions, especially on agricultural issues. Thirdly, in some cases, women may have lower incomes or fewer financial resources as their age increases, which can give men more decision-making power. This finding goes against the findings of several studies, where age was found to positively impact women’s empowerment, and decision-making in agricultural participation and income-generating activities as an increase in age of women often linked with better experience, knowledge, skills, and confidence in asserting their decision-making power to contribute to decision-making processes [34,35,58,59,69]. Potential explanations for our results include social isolation, spousal support and economic vulnerability since widowed and divorced women might be more economically reliant on male family members, thereby limiting their decision-making power. Moreover, married women often have a spouse who can advocate for their rights and support their decision-making. Divorced or widowed women lack this support as they get older, making them more susceptible to losing decision-making power.

Having a better perception of gender equality had a positive effect on WDMA for this segment too. As the score of perception of women towards cultural norms on gender difference was increased by one score, the relative proportion of WDMA increased by 12.45%. It is worth noting that this relative proportion of WDMA increased by only 4% in the first segment. We find that better perception of gender equality has a larger effect on decision-making for women not in marriage compared to women in marriage. The possible reason for this increase is that women who are not in marriage might have more freedom to be aware of gender equality than those who are in marriage. This conclusion aligns with numerous studies highlighting how social norms shape women’s perceptions and decision-making, often resulting in a limittation of their participation in decision-making and achievements [27,36,60].

Our study indicates that wealth is not a guarantee for better participation of women in decision-making about agriculture and their household matters. Compared to women from the poorest household wealth quintile, the women from the poor, middle, rich, and richest household wealth quintiles had decreased relative proportion of WDMA by 79.4%, 64.2%, 74.2%, and 65.3% respectively when we keep the values of other predictors constant. It is worth noting that three more categories of wealth quintiles had a negative association with WDMA in addition to the richest wealth quintile, which was the only significant association in the first segment, compared with the poorest as a reference. The finding is consistent with other studies conducted in Africa and Asia that revealed that wealth does not guarantee women’s empowerment and decision-making [58,65,70]. However, studies in Ethiopia and rural Punjab, India showed that higher household income, women’s personal or earned income, and savings appear to positively influence women’s autonomy [34,62]. The difference might be attributed to the variance in the area of decision-making and the different types of economic status considered.

### Female-led householdss

The third segment formed by BRTM contains female-led households. Only two variables were significantly associated with WDMA: the age of the women and being Orthodox Christianity followers compared to Muslim religious followers.

Similar to the segments of male-led households with either unmarried, divorced, or widowed women, an increase in age had a decreasing effect on decision-making for women from female-led households too. A year increase in the age of the women decreased the relative proportion of WDMA by 1.13%. This could be due to economic dependence as older women might rely more on male family members for economic support, reducing their ability to make decisions about agricultural and household matters despite their label as leaders of their households. In addition, health issues could be a reason that age-related health problems can reduce women’s physical capacity to engage in agricultural activities, leading to a shift in decision-making power to others who can handle these tasks. Moreover, in some rural residents, younger family members are expected to take over leadership roles, which can disempower older women from making decisions.

Contrary to the findings in the first segment, women who were followers of Orthodox Christian religion and who were from female-led households had a higher participation in decision-making than Muslim women who were from female-led households. Orthodox religious followers had an increased relative proportion of WDMA by almost 48% compared to women of Muslim religion. This difference in relative proportion of WDMA between groups was the highest documented. These results could reflect differences in gender roles between different religious groups, in particular among women who are head of households.

In all three segments, neither women’s nor partners’ years of education were found to be significantly associated with WDMA unlike several findings that reported education positively influences women’s decision-making autonomy as the higher education levels improve awareness and understanding of women to ultimately improve women’s authority in decision-making [27,32,68,71]. The reason for the insignificance of years of education of the women, as well as partners, in the current study, might be due to the low education levels of the majority of the participants who spent a few years on education. Furthermore, the mean years of education spent by women was only 1 year and the mean years of study spent by partners was only 2 years. This limited variability might explain the lack of association documented.

### Discussions in terms of Average Marginal Effects (AME) of predictors

AMEs are preferred for interpreting beta regression models as they offer a more intuitive and generalizable understanding of the impact of predictors on the outcome, especially when the relationship is non-linear or influenced by interaction effects. AMEs calculate the marginal effect for each observed value of the predictor variables and average these effects across the sample, providing a comprehensive understanding of the effect, especially in non-linear relationships. These effects offer a precise measure of how a small change in an independent variable directly influences the dependent variable. In simple linear models, marginal effects correspond directly to the estimated regression coefficients, but in non-linear models like Beta regression models, computing marginal effects requires careful consideration. In beta regression, where the outcome is a proportion, AMEs offer a more intuitive interpretation by providing the average change in the predicted proportion for a one-unit change in the predictor, averaged across the sample [72–74]. The next discussion was done in terms of AME for a better interpretation of the estimates and hence advance the lucidity of the findings.

The study examines the effects of various factors on the proportion of WDMA in households. The results show that age has a small impact on WDMA for married women in male-led households, with the proportion increasing by 0.0009 for each additional year of age. As an example, WDMA will increase by 1.8 difference in age of women in this segment. Conversely, for women in female-led households, the proportion of WDMA decreases by 0.0025 with each additional year of age. Similarly, WDMA decrease by 5% for 20 years difference in age of women of female-led household. The study also found that the religion of the women had a significant effect on WDMA, with Orthodox Christianity followers having a lower proportion of WDMA compared to Muslim women in male-led households, while the opposite was true for female-led households. Wealth status is significant in the first and second segments, with WDMA decreasing as household wealth decreases. In the third segment, however, wealth status does not have a significant effect on WDMA. Overall, the study highlights the complex interplay of factors influencing WDMA in households, including age, religion, and wealth status. The study also found that the effect of wealth status on the proportion of WDMA varied depending on the household’s wealth quintile. Specifically, the proportion of WDMA decreased as household wealth increased, with the poorest households having a higher proportion of WDMA compared to the middle and rich households in male-led households. The study also revealed that women’s perceptions of cultural norms regarding gender differences and gender equality, along with participation in local groups, significantly affect WDMA for married women in male-led households. Specifically, each one-point increase in the gender equality score raised WDMA by 0.0096, and participation in social groups raised WDMA by 0.0296. Interpreting the findings in terms of AMEs in the context of women’s decision-making is quite new in the literature and it should also be noted that the possible reasons pointed out in the discussion of the result from BRTM can also apply to this part.

Based on the key findings, the following recommendations are made.

First, interventions should be age-sensitive. Younger women may require tailored support to build autonomy early in their marriage, while programs for older women could focus on overcoming ingrained norms accumulated over time.

Second, awareness-raising activities should target both women and their husbands within existing community groups (e.g., women’s development groups, religious associations, or kebele-level forums). These efforts should aim to shift perceptions of gender-equitable social norms and promote joint household decision-making.

Third, programs must thoughtfully address the complex role of religion. Engaging religious leaders as partners in promoting gender-equitable interpretations of religious teachings could help reduce potential barriers while respecting local cultural and religious contexts.

Finally, and most critically, economic empowerment initiatives alone are insufficient. Although higher household wealth is often associated with better outcomes, our results reveal a masking effect whereby wealthier households might maintain male-dominated decision-making patterns. Therefore, wealth-creation programs (such as microfinance or asset-building schemes) should be explicitly combined with gender-transformative components that challenge intra-household power dynamics rather than assuming economic improvements will automatically translate into greater women’s autonomy.

### Strengths and limitations

The results of this study should be seen in light of the following limitations. The study relied on secondary data from a survey conducted in two regions of Ethiopia that was not specifically designed to assess women’s decision-making autonomy. As a result, important variables, such as detailed intra-household power dynamics, exposure to gender-based violence, and spousal communication patterns, were unavailable. Moreover, the dataset only recorded whether any household member participated in a social group, without identifying the specific participant. Consequently, the analysis could not distinguish a woman’s own participation from that of other household members, which may have different implications for women’s decision-making autonomy. Therefore, we advise readers to interpret the findings for this variable with appropriate caution. Future studies should specifically examine women’s direct participation in social groups. The self-reported nature of the outcome measures may also be subject to social desirability bias.

Despite these limitations, the study offers valuable insights into an important public health topic using robust analytical methods on a large sample. It advances knowledge by incorporating previously underexplored determinants of WDMA.

## Conclusions

The current study sought to identify the determinants of WDMA utilizing a mixed-effects beta regression tree model yielding three distinct and homogeneous household segments from the total heterogeneous households: male-led households with married women, male-led households with single/divorced/widowed women, and female-led households. Contrary to expectations, wealthier households did not improve WDMA, with women from poorer households exhibiting greater autonomy compared to those in higher wealth quintiles. Cultural perceptions of gender norms significantly predicted WDMA in male-led households, regardless of marital status, while women’s age had opposite impacts: positively associated with WDMA for married women in male-led households but negatively for unmarried, divorced, or widowed women and those in female-led households. Religion also played a role, with Orthodox Christian women showing lower WDMA in male-led households when married but higher WDMA in female-led households compared to Muslim women, though it was insignificant for unmarried, divorced, and widowed women in male-led households. Participation in local social groups positively influenced WDMA for married women in male-led households, while the years of education of women and their partners were insignificant most likely due to the generally low educational attainment of the study participants. Future research employing primary quantitative and qualitative approaches is needed to further explore in-depth the mechanisms behind some of the observed associations.

## Supporting information

S1 Text S1 File. Questions employed to measure women’s decision-making autonomy in this study. S2 File. Wealth index and quintiles: Household wealth index and wealth quintile calculation by principal component analysis from the productive assets the households owned during the study periods. S3 File. BC, BR and ML inferences. Beta regression has undergone updates in various ways for different purposes to improve its precise estimation. From those improvements made, the usual maximum likelihood estimation (MLE) has been updated to bias reduction (BR) and bias correction (BC) models to either reduce or correct the bias if bias results from ML, and this needs to be tested to determine if there is any improvement by either BC or BR by either AIC or BIC. In this analysis, comparison indicating of inferences obtained from BC and BR has no difference from the ML and the ML inference was employed as the final result. S4 File.Refitted beta regression models for each segment. This supplementary material contains the BRTM from which separate beta regression models were refitted for each of the segment formed by BRTM. Finally, the average marginal effects (AMEs) of each factor was estimated. S1 Fig. Beta regression model diagnostic tests for beta regression of the first segment. This segment contains 88.2% of the total considered in this study and were households with male-led and married women. S2 Fig. Plot of beta regression tree model of determinants of WDMA in rural Ethiopia. The boxes indicate the predictors of WDMA considered in the current study and the upper two oval nodes were the partitioning variable used in BRTM.
(ZIP)
