# Supplementary material for: Modeling determinants of women’s decision-making autonomy on agriculture and household matters in rural Ethiopia: a mixed effects beta regression tree analysis
Source: PLoS One. 2026 Sep 10;21(9):e0355932. doi: 10.1371/journal.pone.0355932 (PMC13561345; doi:10.1371/journal.pone.0355932)
Supplement: Supplementary file 1 [file pone.0355932.s002.docx]

Dear Authors,

I have carefully read your manuscript on women’s decision-making autonomy (WDMA) in rural Ethiopia. The topic is very relevant for sociology and development studies. Using a "Mixed Effects Beta Regression Tree" is a strong point of this paper, as it captures the complexity of household dynamics better than simpler models. However, I have several concerns that need to be addressed to improve the quality and clarity of the paper.

Major Points:

1. Definitions in the Introduction:

The paper often uses "autonomy" and "empowerment" interchangeably. In sociological literature (e.g., Naila Kabeer, 1999), these concepts are distinct. I suggest the authors provide a clearer definition of "autonomy" in their specific context. Does it mean the woman has the "final say," or is she simply "consulted"? This distinction is crucial for interpreting your results.

2. Local Context:

Ethiopia has diverse cultural and land-tenure systems, especially in Oromia and the Southern regions. These local norms often influence women more than age or wealth. I think the paper needs a small paragraph to explain if these regional and ethnic differences were considered in the sociological analysis.

3. Measuring Participation (Methodology):

The study considers a woman as "participating" if "any family member" is in a social group. I find this to be a weakness. If only the husband is part of a group, it might actually increase his power over the wife rather than increasing her agency. It would be better to focus on the "woman’s own participation."

4. Potential Bias in Interviews:

The manuscript does not mention if the interviews were conducted in private. In rural villages, the presence of a husband or male relatives during the interview can significantly bias a woman’s answers about her decision-making power. This should be mentioned as a limitation.

5. Contradiction in Religion Results (Table 3 vs. Abstract):

There is a serious discrepancy between the Abstract and the Results. In the Abstract, the authors state that being Orthodox (compared to Muslim) is associated with higher autonomy. However, in Table 3 (Node 3), which covers the majority of the sample (n=3961), the coefficient for Orthodox is significantly negative (-0.0468). This indicates lower autonomy for married women in male-led households. Please correct this in the text and discuss why the effect of religion is negative in Node 3 but positive in Node 5.

6. Discussion on Wealth Paradox:

The finding that being in the "Richest" quintile reduces autonomy (-0.0380 in Node 3) is very interesting. Usually, more wealth is expected to increase power, but your data shows the opposite. I suggest discussing this "prestige" factor—do wealthy rural families keep women away from decisions to maintain social status?

Conclusion:
While the statistical work is solid, the paper needs a deeper sociological discussion to explain these patterns. The contradiction regarding the religion variable must be addressed. I recommend a Major Revision.
