# Supplementary material for: Modeling determinants of women’s decision-making autonomy on agriculture and household matters in rural Ethiopia: a mixed effects beta regression tree analysis
Source: PLoS One. 2026 Sep 10;21(9):e0355932. doi: 10.1371/journal.pone.0355932 (PMC13561345; doi:10.1371/journal.pone.0355932)
Supplement: Supplementary file 2 [file pone.0355932.s004.docx]

# **Rebuttal letter**

**PONE-D-25-54211**

**Modeling determinants of women’s decision-making autonomy on agriculture and household matters in rural Ethiopia: a mixed effects beta regression tree analysis**

Dear Editor and referee,

Thank you very much for giving us the opportunity to revise our manuscript. We appreciate all comments and suggestions, which we believe have helped improve the quality of the manuscript. Our responses to the editor’s and reviewers' comments are provided below in a point-by-point format.

Best regards,

Teshome K. Dadi & Co-authors

**Reviewers’ Comment**

**Reviewer #1:**

General Comments

*This manuscript uses a mixed-effects beta regression tree approach to investigate the determinants of women’s decision-making autonomy in rural Ethiopian agriculture and households. The topic is timely and relevant, the methodology appropriate and innovative, and the analysis technically sound. The manuscript is well-organized, clearly written, and the conclusions are data-supported. I recommend minor revisions to improve clarity, interpretation, and presentation.*

**Response:** Thank you very much for your positive and encouraging feedback. We greatly appreciate your recommendation for minor revisions and we have now carefully addressed all comments.

**Major Comments**

*1- Methodological clarity: The description of the statistical model would benefit from additional clarification regarding assumptions, parameter tuning, and validation procedures. A brief justification for selecting this approach over alternative models would improve transparency.*

**Response:** We thank the reviewer for this valuable suggestion. A detailed description of the statistical model, including the underlying theory, mathematical formulas, model assumptions, and selection rationale, was already provided in the “Statistical Analysis” subsection (pages 6–8).

To further improve clarity and transparency as requested, we have added a concise summary of the model and its key assumptions in the body of the manuscript in track change with Latex in black blue color.

*2- Interpretation of results: The discussion could be strengthened by deeper interpretation of the key predictors and their socio-economic implications, with clearer linkage to existing literature.*

**Response:** We believe that our discussion already provides these interpretations and linkage to literature. We believe the discussion is already long and detailed, but we have made some revisions in response to this comment (p. 18).

*3- Policy implications: The policy recommendations are appropriate but could be expanded with more concrete and actionable suggestions.*

**Response:** We have now expanded the policy recommendations (p.22)

**Minor Comments:**

*1- Minor grammatical and typographical errors should be corrected, such as occasional subject–verb agreement issues, inconsistent use of articles (e.g., “a”, “an”, “the”), and minor punctuation errors, mainly in the Introduction and Discussion sections.*

**Response:** Thank you. We have reviewed and addressed grammatical and typographical errors in the manuscript.

*2- Figures and tables would benefit from more detailed captions. For example, in Table X and Figure Y, abbreviations such as “EA”, “HH”, and “DMI” should be clearly defined, and a brief explanation of key variables and units would improve clarity.*

**Response:** We have defined variables with their measurement under the ‘Measurements and operational definitions’ sub section (page 5 to 6). In all the figures and tables also, abbreviations have now been clearly defined.

*3- Organization of the introduction could be slightly improved. For instance, merging the background discussion on women’s empowerment with agricultural decision-making may reduce repetition and enhance logical flow*.

**Response:** We have made some edits in the background section to improve clarity and flow.

*Recommendation
Overall, this is a technically sound and valuable study. I recommend minor revision before acceptance.*

**Response:** We sincerely thank the reviewer for the positive evaluation of our manuscript and for recognizing its technical soundness and scientific value. We greatly appreciate your constructive feedback and recommendation for minor revision.

**Reviewer #2:**

*The work is good and can be published after addressing the minor comments.*

**Response:** We sincerely thank the reviewer for the positive assessment of our work and for recommending its publication.

1. *Revise the abstract mentioning the main work and main outcomes.*

**Response:** The abstract already summarizes the main methodology employed and the key factors associated with women's decision-making autonomy in heterogeneous households. We would also like to note that the journal has a strict word limit for the abstract, which limits the amount of detail that can be included.

2. *Look the whole manuscript regarding the spelling and grammatical errors and fix them.*

**Response: We would like to thank the reviewer for this** helpful suggestion. We have now checked the manuscript for spelling and grammatical error.

3. *At the end of the introduction write the novelty of the current work clearly*

**Response: Thank you very much. We updated the introduction section by stating the novelty of our work**

4. *Cite the references properly where from the authors got the idea.*

**Response: Thank you. We have gone through the reference list and have updated citation which was incorrect.**

5. *What are the limitations and future recommendations of the current work in the end of the conclusion section?*

**Response:** Thank you for this valuable suggestion. We have included limitations and provided recommendations for future studies to build upon this work.

**Reviewer #3:**

*The authors made a meaningful methodological contribution by applying a mixed-effects beta regression tree to analyze WDMA in rural Ethiopia. I suggest the followings to improve this study:*

**Response:** Thank you for your encouragement!

*- Report intra-class correlation (ICC).*

**Response: We have reported the ICC under the sub section of Survey Round on page #6.**

*- Provide reliability of data by apply any suitable test e.g., Cronbach’s alpha test.*

**Response: We have calculated the** Cronbach’s alpha test and reported in the revised manuscript (p. 5)

*- Provide cross-validation for tree model.*

**Response:** We thank the reviewer for raising this point. In this study, it was through **parameter stability test** that the robustness of the **Beta Regression Tree** model was evaluated. This process confirmed that the main findings regarding the determinants of women’s decision-making autonomy were stable and not overly sensitive to specific parameter choices. Although cross-validation is a valuable tool for assessing predictive performance and guarding against over fitting, our primary objective was **interpretive inference** on the complex relationships and interactions within the data, rather than pure predictive accuracy. Given the bounded nature of the outcome variable and the clustered survey design, we believe the parameter stability analysis provided sufficient evidence of model reliability.

*- Distinguish agricultural vs household autonomy if conceptually separable.*

**Response:** Thank you for your suggestion. They are not conceptually separable in the context of this study.

**Reviewer #4:**

*Dear Authors,*

*I have carefully read your manuscript on women’s decision-making autonomy (WDMA) in rural Ethiopia. The topic is very relevant for sociology and development studies. Using a "Mixed Effects Beta Regression Tree" is a strong point of this paper, as it captures the complexity of household dynamics better than simpler models. However, I have several concerns that need to be addressed to improve the quality and clarity of the paper.*

**Response:** We sincerely thank the reviewer for the careful and thoughtful evaluation of our manuscript and for recognizing the relevance of our study, particularly to the fields of sociology and development studies, where understanding women's decision-making autonomy is of considerable importance. We are especially grateful for the reviewer's recognition of the methodological strength of our study, particularly the application of the Mixed Effects Beta Regression Tree model to capture the complexity and heterogeneity of household decision making.

**Major Points:**

1. Definitions in the Introduction:

*The paper often uses "autonomy" and "empowerment" interchangeably. In sociological literature (e.g., Naila Kabeer, 1999), these concepts are distinct. I suggest the authors provide a clearer definition of "autonomy" in their specific context. Does it mean the woman has the "final say," or is she simply "consulted"? This distinction is crucial for interpreting your results.*

**Response:** Thank you for the feedback. Regarding autonomy and empowerment, we would like to clarify that we did not use the two interchangeably. We fully acknowledge that these are related but conceptually distinct constructs, and we have intentionally avoided using them interchangeably throughout the manuscript. Consistent with the literature, we recognize that **women's decision-making autonomy is one of the key domains of women's empowerment,** rather than a synonym for empowerment itself. We also acknowledge that some previous studies have used women's decision-making autonomy as a proxy or indicator of women's empowerment, and we have cited this literature where appropriate. Nevertheless, in our study, we have maintained a clear conceptual distinction between the two constructs and have consistently focused on **women's decision-making autonomy** as the outcome of interest. To further clarify this point, we have made minor edits to the wording to ensure that the distinction is clear and unambiguous to readers.

The second concern is about the meaning of autonomy. The contextual definition of autonomy and its measurements in our study was described in ‘Measurements and operational definitions’ sub section of the method section on page 5. In this study, women's decision-making autonomy refers to a woman's **having the final say**, either independently or jointly with her husband/partner, on household and agricultural decisions, consistent with the definition used in the survey instrument and previous studies. We have addressed this concern in the body of the manuscript on page 5.

*2. Local Context:
Ethiopia has diverse cultural and land-tenure systems, especially in Oromia and the Southern regions. These local norms often influence women more than age or wealth. I think the paper needs a small paragraph to explain if these regional and ethnic differences were considered in the sociological analysis.*

**Response:** We thank the reviewer for this valuable observation. The present study was however based on secondary data that were not originally collected to specifically investigate the sociological effects of cultural norms, ethnicity, or land-tenure systems on women's decision-making autonomy. Thus, these variables were not available for analysis and could not be explicitly examined in our models. We have acknowledged this limitation in the **Limitations** section of the revised manuscript and recommend that future studies incorporate detailed information for further exploration of the potential predictors that could influence women's decision-making autonomy.

3. *Measuring Participation (Methodology):*

*The study considers a woman as "participating" if "any family member" is in a social group. I find this to be a weakness. If only the husband is part of a group, it might actually increase his power over the wife rather than increasing her agency. It would be better to focus on the "woman’s own participation."*

**Response:** We thank the reviewer for this insightful comment. We agree that **a woman's own participation** in social groups is conceptually more relevant to women's decision-making autonomy than the participation of any household member. However, this study was based on secondary data, and the available variable only indicated whether **any household member** participated in a social group, without identifying which household member was involved. In the previous version of the manuscript, we interpreted this variable under the assumption that participation by any household member could facilitate information diffusion and potentially enhance women's decision-making autonomy. We acknowledge the reviewer's important point that, if the participant was the husband or another household member, such participation may instead reinforce existing household power dynamics and may not necessarily improve, and could even reduce a woman's autonomy. In light of this valuable observation, we have revised the manuscript to explicitly acknowledge this limitation in the **Limitations** sections and advise readers to interpret the findings for this variable with appropriate caution. Future studies should distinguish **women's own participation** from that of other household members to better assess its influence on women's decision-making autonomy.

*4. Potential Bias in Interviews:*

*The manuscript does not mention if the interviews were conducted in private. In rural villages, the presence of a husband or male relatives during the interview can significantly bias a woman’s answers about her decision-making power. This should be mentioned as a limitation.*

**Response:** We are thankful about the comment and we acknowledge that interview privacy is an important consideration when collecting data on sensitive topics such as women's decision-making autonomy. However, the present study was based on secondary data. The survey was conducted in accordance with established ethical standards, including obtaining informed consent and ensuring participants' confidentiality, as documented in the survey protocol. The dataset does not provide information on the interview setting (e.g., whether interviews were conducted in private), and we therefore cannot comment further on this aspect but we have no reason to suspect that the interviews were not conducted in private. We have no evidence to suggest that the quality of the data was compromised by the presence of other household members during the interviews.

*5. Contradiction in Religion Results (Table 3 vs. Abstract):*

*There is a serious discrepancy between the Abstract and the Results. In the Abstract, the authors state that being Orthodox (compared to Muslim) is associated with higher autonomy. However, in Table 3 (Node 3), which covers the majority of the sample (n=3961), the coefficient for Orthodox is significantly negative (-0.0468). This indicates lower autonomy for married women in male-led households. Please correct this in the text and discuss why the effect of religion is negative in Node 3 but positive in Node 5.*

**Response: Thank you for this important picking of the typographic error we made in the abstract, which we have now corrected.**

*6. Discussion on Wealth Paradox:*

*The finding that being in the "Richest" quintile reduces autonomy (-0.0380 in Node 3) is very interesting. Usually, more wealth is expected to increase power, but your data shows the opposite. I suggest discussing this "prestige" factor—do wealthy rural families keep women away from decisions to maintain social status?*

Response: Thank you for this comment, which we have now incorporated in the manuscript on page 18.

*Conclusion:
While the statistical work is solid, the paper needs a deeper sociological discussion to explain these patterns. The contradiction regarding the religion variable must be addressed. I recommend a Major Revision.*

**Response:** We thank the reviewer and we have addressed the valuable comments provided.

________________________________________

***Reviewer #5:***

*The manuscript addresses an important and relevant topic concerning women’s decision-making autonomy in agricultural and household contexts in rural Ethiopia. The use of a relatively large dataset and the attempt to capture heterogeneity through a mixed-effects beta regression tree model are notable strengths of the study.*

**Response:** We greatly appreciate your recognition of the importance of the research topic, the use of a large nationally representative dataset, and the application of the mixed-effects beta regression tree model to capture heterogeneity in women's decision-making autonomy. Your thoughtful feedback is greatly valued and has helped us further improve the manuscript.

*However, several issues should be addressed to improve the quality and clarity of the manuscript:*

*Statistical clarity and justification:*

*While the use of the beta regression tree model is innovative and appropriate for bounded outcomes and heterogeneous data, the manuscript lacks sufficient justification for selecting this model over alternative approaches. The discussion of model selection (based primarily on AIC) should be expanded, and more information on model diagnostics and goodness-of-fit should be provided.*

**Response:** In our manuscript, we have reported that parameter instability test was one of the testing method in addition to the AIC. The model diagnostic and goodness-of-fit test was also done through parameter stability test which was reported from pages 8-9 of the manuscript.

*Interpretation of findings:*

*Some findings appear counterintuitive, particularly the negative association between household wealth and women’s decision-making autonomy in certain subgroups. These results require deeper theoretical engagement and clearer contextual explanation. Strengthening the linkage between empirical findings and existing literature would enhance the contribution of the study.*

**Response:** We have discussed these results further and suggested possible explanations for the results. We have made linkage to existing literature as much as possible.

*Data availability and transparency:*

*The data underlying the findings are not fully publicly available and require access through a third party. While ethical constraints may justify this, the data availability statement should provide clearer details on the procedure for requesting access and the conditions under which the data can be obtained. This would improve transparency and alignment with journal policies.*

**Response:** We will update and explain the procedures in the submission system.

*Organization and presentation:*

*Some sections—particularly the methods and discussion—are overly detailed and could benefit from improved structure and conciseness. Reducing redundancy and focusing on key analytical steps would improve readability.*

**Response:** Thank you very much! We also received valuable feedback from other reviewers recommending a more detailed description of certain methodological procedures and a more comprehensive discussion of some significant findings. In revising the manuscript, we carefully considered all reviewers' comments and sought to strike an appropriate balance among the various recommendations.

*Language and readability:*

*The manuscript is generally understandable; however, it would benefit from language editing to improve clarity, reduce repetition, and refine sentence structure. Several sections contain long and complex sentences that may hinder comprehension.*

**Response:** Thank you for your valuable feedback. We have done our best to address these concerns through careful editing of the manuscript.

*Overall, the manuscript has merit and contributes to the literature on women’s empowerment and decision-making autonomy. With revisions addressing the points above, the paper could be strengthened significantly in terms of clarity, rigor, and interpretability.*

**Response:** We thank you very much!
