# Supplementary material for: Modeling determinants of women’s decision-making autonomy on agriculture and household matters in rural Ethiopia: a mixed effects beta regression tree analysis
Source: PLoS One. 2026 Sep 10;21(9):e0355932. doi: 10.1371/journal.pone.0355932 (PMC13561345; doi:10.1371/journal.pone.0355932)
Supplement: Supplementary file 3 [file pone.0355932.s001.zip › Supplementaries/S1_File.pdf]

Questions used to measure women's decision-making autonomy in the agriculture-nutrition study

ENUMERATOR: Ask G01 for all categories of activities before asking G02.<br><br>If the household does not engage in that particular activity, enter code for "Decision not made" and proceed to the next activity.		When decisions are made regarding the following aspects of household life, who typically makes the decisions?<br>If response = option 2 ["Main female or Wife], go to **next domain**<br>Otherwise >>other section<br>**CODE 1**
		**G01**
**A**	Agricultural production?	
**B**	What inputs to buy for agricultural production?	
**C**	What types of crops to grow for agricultural production?	
**D**	Who would take crops to the market for sale?	
**E**	Livestock raising?	
**F**	Non-farm business activity (For example: shop owner, animal trading, wood and charcoal selling, handicrafts, etc.,	
**G**	Your own (singular) wage or salary employment?	
**H1**	Major household expenditures? (Such as livestock purchase, rent land)	
**H2**	Minor household expenditures? (Such as food for daily consumption or other household needs, like clothes)	
**M**	Whether or not to use family planning to space or limit births?	
**N**	Homestead gardening?	

CODE1: (G01) Decision making

Main male or husband ....…………1

Main female or wife/self ....…………2

Husband and wife jointly ....…………3

Someone else in the household...................…………4

Jointly with others inside the household......…………5

Jointly with someone else outside the household……...6

Someone outside the household/other ....…………7

Decision not made because not applicable ....………...98
