# Supplementary material for: Modeling determinants of women’s decision-making autonomy on agriculture and household matters in rural Ethiopia: a mixed effects beta regression tree analysis
Source: PLoS One. 2026 Sep 10;21(9):e0355932. doi: 10.1371/journal.pone.0355932 (PMC13561345; doi:10.1371/journal.pone.0355932)
Supplement: Supplementary file 3 [file pone.0355932.s001.zip › Supplementaries/S2_File.pdf]

Determination of rural household wealth index and wealth quintiles

Our study has been conducted in the rural community of the former SNNP and Oromia regional states of Ethiopia. To quantify the economic status of the households, the calculation of household wealth index and ultimately household wealth quintiles from the rural Productive Assets (PA) by the Principal Component Analysis (PCA). The quantity of PA that each household owned was originally asked and recorded for 17 items in the questionnaire. Then, each item has been changed into binary as yes (present) or no (absent), which were coded as '1' and '0', respectively (Changed to 0 if they have no item, and 1 if they have at least one item of the PA). After the type of response changed from quantitative to binary, frequency analyses were done to sort out variables that were rare (percentage ≤ 5%) and common (percentage >95%), since the rare and common PAs did not help differentiate the wealth status of households. Meaning, the percentage of households owning the items should be within the range of [5%, 95%] to be included in the wealth quintile calculation. All data processing regarding the wealth index or quintiles was done by Stata statistical software. The frequency table below was generated from the Stata command to determine frequency (percent) with the frequency of missing values for those who own hoes with variable name 'pa1_hoe' as one example, and this frequency analysis was done for all 17 items of PAs.

tab pa1_hoe, m

Table 1: Percentage of owning hoe by the households included in the study

Does the household currently owned any hoe?	Frequency	Percent	Cumulative Frequency
No	2,960	62.94	62.94
Yes	1,738	**36.96**	99.89
Missing	5	0.11	100
Total	4,703	100	

In Table 1, the percentage of households owned hoe is 36.96% and is within the acceptable range of 5% and 95%. Therefore, hoe would be included in to the analysis. We did same for other items and included all the items satisfying this condition.

As can be visualized from Table 2, 1) Hoe, 2) Plough materials, 3) Saw, 4) Hammer, 5) Sickle, 6) Grain storage, 7) Grain sacks, 8) Axe, 9) Pick-axe, 10) Saddle bags for donkeys, 11) Saddle bags for horses/mules, 12) Beehive, 13) Chicken coop, and 14) Pesticide sprayer assets were considered for the calculation of the household wealth quintiles. Refer to Table 2 for details.

Table 2: Summary of percentages of owning items by the households

✓=Criteria is satisfied, X=Not satisfied to be included in the calculation, '.' =missing

	TYPE OF PRODUCTIVE ASSET	Variable Name no (absent)=0, yes (present) =1	Percentages (%)			Remarks
			No	Yes	.	
1.	Hoe (zabia/chikaro)	pa1_hoe	62.94	36.96	0.11	✓
2.	Plough materials (mofer, kenber)	pa2_plough	32.19	67.70	0.11	✓
3.	Fas	pa3_faz	96.39	3.51	0.11	X
4.	Saw (megaz)	pa4_saw	88.43	11.46	0.11	✓
5.	Hammer or martelo	pa5_hamm	88.84	11.06	011	✓
6.	Sickle (machid)	pa6_sickle	24.26	75.63	0.11	✓
7.	Grain storage (gotara)	pa8_gstorage	57.90	41.99	0.11	✓
8.	Grain sacks (chiret or kasha)	pa9_gsacks	19.31	80.59	0.11	✓
9.	Axe (metrebia)	pa10_axe	30.11	69.79	0.11	✓
10.	Pick-axe (doma)	pa11_pickaxe	87.41	12.48	0.11	✓
11.	Saddle bags/boxes for donkey	pa12_sadbagd	87.18	12.69	0.13	✓
12.	Saddle for Horse/Mules (Korecha)	pa13_sadbagh	84.48	15.42	0.11	✓
13.	Beehive	pa14_beehiv	70.00	29.90	0.11	✓
14.	Honey extractor	pa15_honext	98.70	1.19	0.11	X
15.	Cart	pa16_cart	96.45	3.44	0.11	X
16.	Chicken coop	pa17_chickencp	94.62	5.27	0.11	✓
17.	Pesticide sprayer	pa18_pestspry	92.83	7.06	0.11	✓

Next, Principal Component Analysis (PCA) was employed followed by predicting the wealth index score by these 14 items satisfied the criteria. Refer to stata command for PCA with its output.

Table 3: Output of Principal Component Analysis

pca *pa1_hoe pa2_plough pa3_faz pa5_hamm pa6_sickle pa8_gstorage pa9_gsacks pa10_axe pa11_pickaxe pa12_sadbagd pa13_sadbagh pa17_chickencp pa18_pestspry*

```
. pca pa1_hoe pa2_plough pa3_faz pa5_hamm pa6_sickle pa8_gstorage pa9_gsacks pa10_axe pa11_pickaxe pa12_sadbagd pa13_sad
> bagh pa17_chickencp pa18_pestspry

Principal components/correlation                 Number of obs    =      4,697
                                                 Number of comp.  =         13
                                                 Trace            =  [illegible]
    Rotation: (unrotated = principal)            Rho              =     1.0000
```

Component	Eigenvalue	Difference	Proportion	Cumulative
Comp1	2.48402	1.24608	0.1911	0.1911
Comp2	1.23793	.149122	0.0952	0.2863
Comp3	1.08881	.0420338	0.0838	0.3701
Comp4	1.04678	.0750842	0.0805	0.4506
Comp5	.971693	.0717143	0.0747	0.5253
Comp6	.899979	.0375637	0.0692	0.5946
Comp7	.862415	.0189804	0.0663	0.6609
Comp8	.843435	.0339335	0.0649	0.7258
Comp9	.809501	.0354381	0.0623	0.7880
Comp10	.774063	.0826075	0.0595	0.8476
Comp11	.691456	.0213983	0.0532	0.9008
Comp12	.670058	.0501966	0.0515	0.9523
Comp13	.619861	.	0.0477	1.0000

Principal components (eigenvectors)

Variable	Comp1	Comp2	Comp3	Comp4	Comp5	Comp6	Comp7	Comp8	Comp9	Comp10
pa1_hoe	0.2598	-0.1037	0.1816	0.6309	-0.2528	-0.0904	-0.1582	0.0718	-0.0520	0.2060
pa2_plough	0.3203	-0.3387	-0.1646	-0.0295	0.3370	0.0525	-0.2557	0.0797	-0.3924	-0.1141
pa3_faz	0.2161	0.3137	-0.1634	0.0194	-0.0142	0.8006	-0.0906	0.3848	0.1177	0.1208
pa5_hamm	0.2794	0.4729	0.0741	0.1072	-0.3107	-0.1619	-0.1135	-0.2913	0.0658	0.1732
pa6_sickle	0.3432	-0.2633	0.2420	-0.1802	-0.1417	0.1179	-0.0607	-0.0525	0.1752	-0.6052
pa8_gstorage	0.2886	-0.2812	-0.4464	-0.0080	0.2636	-0.1447	-0.1956	-0.1089	0.1813	0.4446
pa9_gsacks	0.2833	-0.1893	0.3137	-0.1374	-0.1144	0.0171	0.5840	0.2382	-0.4237	0.3397
pa10_axe	0.3581	-0.2812	0.1917	-0.0189	-0.2183	0.1515	-0.0894	-0.2809	0.2886	0.0433
pa11_pickaxe	0.2153	0.2143	0.3756	-0.2916	0.2748	-0.3861	-0.2194	0.5175	0.3327	0.1072
pa12_sadbagd	0.2539	0.0202	-0.4807	0.1549	-0.0670	-0.2178	0.5774	0.1994	0.3748	-0.2335
pa13_sadbagh	0.2630	0.2551	-0.1638	-0.5668	-0.0899	0.0010	0.0704	-0.3701	-0.1488	0.1214
pa17_chick~p	0.1500	0.2143	0.2950	0.2796	0.6977	0.1537	0.2992	-0.3886	0.0927	-0.0481
pa18_pests~y	0.3050	0.3631	-0.1693	0.1699	0.0249	-0.2093	-0.1466	0.0990	-0.4657	-0.3684

Variable	Comp11	Comp12	Comp13	Unexplained
pa1_hoe	0.5327	0.2388	-0.0494	0
pa2_plough	0.1633	-0.5250	-0.3121	0
pa3_faz	0.0013	0.0137	0.0430	0
pa5_hamm	-0.1354	-0.6101	0.1894	0
pa6_sickle	0.1527	0.0000	0.5111	0
pa8_gstorage	-0.1675	0.1758	0.4563	0
pa9_gsacks	-0.1730	-0.0290	0.1725	0
pa10_axe	-0.4514	0.1666	-0.5305	0
pa11_pickaxe	0.0491	0.0322	-0.1244	0
pa12_sadbagd	0.0980	-0.1170	-0.1913	0
pa13_sadbagh	0.4800	0.2667	-0.1709	0
pa17_chick~p	0.0439	0.0464	0.0202	0
pa18_pests~y	-0.3766	0.3840	0.0062	0

As it can be read from the above table, 13 components were exhaustively explained the variation with no unexplained values. From these 13 components, we calculated the predicted value of the wealth index score with the Stata command ***predict wis***; where 'wis' is the variable name for wealth index score, and the summary statistics are:

Table 4: Summary statistics of predicted wealth index score

VARIABLE	OBS	MEAN	STD. DEV.	MIN	MAX
**WIS**	4,697	4.32e-09	1.576076	-3.188328	6.401171

Then, the households were grouped in to 5 wealth quintiles by the below Stata command and labeled from the poorest to the richest.

xtile wealth_quintile= wis, nq(5)

label define wi 1 "Poorest" 2 "Poor" 3 "Middle" 4 "Rich" 5 "Richest"

label values wealth_quintile wi

label list wealth_quintile

```
. tab wealth_quintile,m
```

5 quantiles of wis	Freq.	Percent	Cum.
Poorest	960	20.41	20.41
Poor	930	19.77	40.19
Middle	935	19.88	60.07
Rich	934	19.86	79.93
Richest	938	19.94	99.87
.	6	0.13	100.00
Total	4,703	100.00	
