# Supplementary material for: Modeling determinants of women’s decision-making autonomy on agriculture and household matters in rural Ethiopia: a mixed effects beta regression tree analysis
Source: PLoS One. 2026 Sep 10;21(9):e0355932. doi: 10.1371/journal.pone.0355932 (PMC13561345; doi:10.1371/journal.pone.0355932)
Supplement: Supplementary file 3 [file pone.0355932.s001.zip › Supplementaries/S3_File.pdf]

Comparisons of ML, BR, and BC Beta Regression models by AIC & BIC

Beta regression has undergone updates in various ways for different purposes to improve its precise estimation. From those improvements made, the usual maximum likelihood estimation (MLE) has been updated to bias reduction (BR) and bias correction (BC) models to either reduce or correct the bias if bias results from ML, and this needs to be tested to determine if there is any improvement by either BC or BR by either AIC or BIC.

```
Beta.ML<-betareg(trans_wdma~FS+WE+PE+Age+CN+RW+WQ+SP+SR+MS+GH|woreda+SR, link
= "logit",type = "ML", data = wdma2)
summary(Beta.ML)
```

```
##
## Call:
## betareg(formula = trans_wdma ~ FS + WE + PE + Age + CN + RW + WQ + SP +
##     SR + MS + GH | woreda + SR, data = wdma2, link = "logit", type = "ML")
##
## Quantile residuals:
##     Min      1Q  Median      3Q     Max
## -7.3370 -0.2463  0.0440  0.2779  6.3810
##
## Coefficients (mean model with logit link):
##               Estimate Std. Error z value Pr(>|z|)
## (Intercept) -0.7014772  0.1087645  -6.450 1.12e-10 ***
## FS          -0.0121177  0.0079208  -1.530 0.126054
## WE           0.0014374  0.0070491   0.204 0.838424
## PE           0.0070146  0.0060054   1.168 0.242793
## Age          0.0002587  0.0015454   0.167 0.867059
## CN           0.0317053  0.0047083   6.734 1.65e-11 ***
## RW2         -0.1498739  0.0392399  -3.819 0.000134 ***
## RW3          0.0196903  0.0488136   0.403 0.686671
## WQ2         -0.0292598  0.0483330  -0.605 0.544927
## WQ3         -0.0527297  0.0493185  -1.069 0.284995
## WQ4         -0.0803777  0.0482390  -1.666 0.095666 .
## WQ5         -0.1961371  0.0494491  -3.966 7.30e-05 ***
## SP1          0.1280488  0.0446040   2.871 0.004094 **
## SR1          0.1550563  0.0367308   4.221 2.43e-05 ***
## MS2          0.0376204  0.0955375   0.394 0.693747
## MS3          0.5910420  0.1131855   5.222 1.77e-07 ***
## GH1          2.2671489  0.0959461  23.629  < 2e-16 ***
##
## Phi coefficients (precision model with log link):
##             Estimate Std. Error z value Pr(>|z|)
## (Intercept)  0.68292    0.06439  10.606  < 2e-16 ***
## woreda2     -0.37885    0.07597  -4.987 6.14e-07 ***
## woreda3     -0.27308    0.08029  -3.401 0.000671 ***
## woreda4     -0.68927    0.07656  -9.003  < 2e-16 ***
## woreda5     -0.58944    0.08696  -6.779 1.21e-11 ***
## woreda6      0.12879    0.08779   1.467 0.142376
## woreda7      0.46585    0.08315   5.603 2.11e-08 ***
```

```
## woreda8      0.48869    0.07030   6.952 3.60e-12 ***
## woreda9     -0.02181    0.07859  -0.278 0.781384
## SR1          0.42822    0.04007  10.686  < 2e-16 ***
## ---
## Signif. codes:  0 '***' 0.001 '**' 0.01 '*' 0.05 '.' 0.1 ' ' 1
##
## Type of estimator: ML (maximum likelihood)
## Log-likelihood:  1399 on 27 Df
## Pseudo R-squared: 0.2482
## Number of iterations: 38 (BFGS) + 3 (Fisher scoring)
```

```
Beta.ML<-betareg(trans_wdma~FS+WE+PE+Age+CN+RW+WQ+SP+SR+MS+GH|woreda+SR, link
= "logit",type = "ML", data = wdma2)
summary(Beta.ML)
```

```
##
## Call:
## betareg(formula = trans_wdma ~ FS + WE + PE + Age + CN + RW + WQ + SP +
##     SR + MS + GH | woreda + SR, data = wdma2, link = "logit", type = "ML")
##
## Quantile residuals:
##     Min      1Q  Median      3Q     Max
## -7.3370 -0.2463  0.0440  0.2779  6.3810
##
## Coefficients (mean model with logit link):
##               Estimate Std. Error z value Pr(>|z|)
## (Intercept) -0.7014772  0.1087645  -6.450 1.12e-10 ***
## FS          -0.0121177  0.0079208  -1.530 0.126054
## WE           0.0014374  0.0070491   0.204 0.838424
## PE           0.0070146  0.0060054   1.168 0.242793
## Age          0.0002587  0.0015454   0.167 0.867059
## CN           0.0317053  0.0047083   6.734 1.65e-11 ***
## RW2         -0.1498739  0.0392399  -3.819 0.000134 ***
## RW3          0.0196903  0.0488136   0.403 0.686671
## WQ2         -0.0292598  0.0483330  -0.605 0.544927
## WQ3         -0.0527297  0.0493185  -1.069 0.284995
## WQ4         -0.0803777  0.0482390  -1.666 0.095666 .
## WQ5         -0.1961371  0.0494491  -3.966 7.30e-05 ***
## SP1          0.1280488  0.0446040   2.871 0.004094 **
## SR1          0.1550563  0.0367308   4.221 2.43e-05 ***
## MS2          0.0376204  0.0955375   0.394 0.693747
## MS3          0.5910420  0.1131855   5.222 1.77e-07 ***
## GH1          2.2671489  0.0959461  23.629  < 2e-16 ***
##
## Phi coefficients (precision model with log link):
##             Estimate Std. Error z value Pr(>|z|)
## (Intercept)  0.68292    0.06439  10.606  < 2e-16 ***
## woreda2     -0.37885    0.07597  -4.987 6.14e-07 ***
## woreda3     -0.27308    0.08029  -3.401 0.000671 ***
## woreda4     -0.68927    0.07656  -9.003  < 2e-16 ***
```

```
## woreda5     -0.58944    0.08696  -6.779 1.21e-11 ***
## woreda6      0.12879    0.08779   1.467 0.142376    
## woreda7      0.46585    0.08315   5.603 2.11e-08 ***
## woreda8      0.48869    0.07030   6.952 3.60e-12 ***
## woreda9     -0.02181    0.07859  -0.278 0.781384    
## SR1          0.42822    0.04007  10.686  < 2e-16 ***
## ---
## Signif. codes:  0 '***' 0.001 '**' 0.01 '*' 0.05 '.' 0.1 ' ' 1
## 
## Type of estimator: ML (maximum likelihood)
## Log-likelihood:  1399 on 27 Df
## Pseudo R-squared: 0.2482
## Number of iterations: 38 (BFGS) + 3 (Fisher scoring)
```

```
Beta.BC<-betareg(trans_wdma~FS+WE+PE+Age+CN+RW+WQ+SP+SR+MS+GH|woreda+SR, link
= "logit",type = "BC", data = wdma2)
summary(Beta.BC)
```

```
## 
## Call:
## betareg(formula = trans_wdma ~ FS + WE + PE + Age + CN + RW + WQ + SP + 
##     SR + MS + GH | woreda + SR, data = wdma2, link = "logit", type = "BC")
## 
## Quantile residuals:
##     Min      1Q  Median      3Q     Max 
## -7.3138 -0.2458  0.0439  0.2771  6.3638 
## 
## Coefficients (mean model with logit link):
##               Estimate Std. Error z value Pr(>|z|)    
## (Intercept) -0.7010682  0.1089531  -6.435 1.24e-10 ***
## FS          -0.0121040  0.0079344  -1.526 0.127129    
## WE           0.0014440  0.0070605   0.205 0.837948    
## PE           0.0070075  0.0060161   1.165 0.244102    
## Age          0.0002583  0.0015480   0.167 0.867461    
## CN           0.0316882  0.0047163   6.719 1.83e-11 ***
## RW2         -0.1497611  0.0393051  -3.810 0.000139 ***
## RW3          0.0196406  0.0489054   0.402 0.687975    
## WQ2         -0.0292174  0.0484147  -0.603 0.546188    
## WQ3         -0.0526530  0.0494013  -1.066 0.286504    
## WQ4         -0.0802862  0.0483239  -1.661 0.096629 .  
## WQ5         -0.1959705  0.0495344  -3.956 7.61e-05 ***
## SP1          0.1279544  0.0446847   2.863 0.004190 ** 
## SR1          0.1549007  0.0367884   4.211 2.55e-05 ***
## MS2          0.0360189  0.0956397   0.377 0.706464    
## MS3          0.5926363  0.1133376   5.229 1.70e-07 ***
## GH1          2.2686000  0.0960395  23.622  < 2e-16 ***
## 
## Phi coefficients (precision model with log link):
```

```
##              Estimate Std. Error z value Pr(>|z|)
## (Intercept)  0.67593    0.06435  10.505  < 2e-16 ***
## woreda2     -0.37682    0.07593  -4.963 6.95e-07 ***
## woreda3     -0.27177    0.08024  -3.387 0.000707 ***
## woreda4     -0.68684    0.07652  -8.976  < 2e-16 ***
## woreda5     -0.58857    0.08690  -6.773 1.26e-11 ***
## woreda6      0.12891    0.08773   1.469 0.141757
## woreda7      0.46532    0.08310   5.600 2.15e-08 ***
## woreda8      0.49005    0.07025   6.975 3.05e-12 ***
## woreda9     -0.02136    0.07854  -0.272 0.785672
## SR1          0.42877    0.04005  10.705  < 2e-16 ***
## ---
## Signif. codes:  0 '***' 0.001 '**' 0.01 '*' 0.05 '.' 0.1 ' ' 1
##
## Type of estimator: BC (bias-corrected)
## Log-likelihood:  1399 on 27 Df
## Pseudo R-squared: 0.2482
## Number of iterations: 38 (BFGS) + 3 (Fisher scoring)
```

```
Beta.BR<-betareg(trans_wdma~FS+WE+PE+Age+CN+RW+WQ+SP+SR+MS+GH|woreda+SR, link
= "logit",type = "BR", data = wdma2)
summary(Beta.BR)
```

```
##
## Call:
## betareg(formula = trans_wdma ~ FS + WE + PE + Age + CN + RW + WQ + SP +
##     SR + MS + GH | woreda + SR, data = wdma2, link = "logit", type = "BR")
##
## Quantile residuals:
##     Min      1Q  Median      3Q     Max
## -7.3136 -0.2458  0.0439  0.2770  6.3636
##
## Coefficients (mean model with logit link):
##                Estimate Std. Error z value Pr(>|z|)
## (Intercept) -0.7011480  0.1089535  -6.435 1.23e-10 ***
## FS          -0.0121128  0.0079344  -1.527 0.126855
## WE           0.0014542  0.0070605   0.206 0.836825
## PE           0.0069976  0.0060161   1.163 0.244766
## Age          0.0002593  0.0015480   0.168 0.866956
## CN           0.0316919  0.0047164   6.720 1.82e-11 ***
## RW2         -0.1496410  0.0393047  -3.807 0.000141 ***
## RW3          0.0196941  0.0489047   0.403 0.687166
## WQ2         -0.0292556  0.0484151  -0.604 0.545666
## WQ3         -0.0527019  0.0494016  -1.067 0.286060
## WQ4         -0.0802917  0.0483242  -1.662 0.096608 .
## WQ5         -0.1959433  0.0495347  -3.956 7.63e-05 ***
## SP1          0.1279398  0.0446854   2.863 0.004195 **
## SR1          0.1549865  0.0367887   4.213 2.52e-05 ***
## MS2          0.0356557  0.0956397   0.373 0.709288
## MS3          0.5926592  0.1133380   5.229 1.70e-07 ***
```

```
## GH1          2.2688174  0.0960397  23.624  < 2e-16 ***
##
## Phi coefficients (precision model with log link):
##              Estimate Std. Error z value Pr(>|z|)
## (Intercept)  0.67589    0.06435  10.504  < 2e-16 ***
## woreda2     -0.37685    0.07593  -4.963 6.93e-07 ***
## woreda3     -0.27166    0.08024  -3.386  0.00071 ***
## woreda4     -0.68687    0.07652  -8.977  < 2e-16 ***
## woreda5     -0.58863    0.08690  -6.773 1.26e-11 ***
## woreda6      0.12888    0.08773   1.469  0.14183
## woreda7      0.46519    0.08310   5.598 2.17e-08 ***
## woreda8      0.49007    0.07025   6.976 3.04e-12 ***
## woreda9     -0.02108    0.07854  -0.268  0.78834
## SR1          0.42878    0.04005  10.706  < 2e-16 ***
## ---
## Signif. codes:  0 '***' 0.001 '**' 0.01 '*' 0.05 '.' 0.1 ' ' 1
##
## Type of estimator: BR (bias-reduced)
## Log-likelihood:  1399 on 27 Df
## Pseudo R-squared: 0.2482
## Number of iterations: 38 (BFGS) + 11 (quasi Fisher scoring)
```

Model Comparison by Akaki Information Criteria (AIC) and Bayesian Information Criteria (BIC)

As it was shown above, the three models are non-nested models and hence AIC or BIC can be used to choose the best model of all. The lower AIC/BIC values indicate a better balance between model fit and complexity. When comparing non-nested models, the model with the lowest AIC value is generally considered the best-fitting model. In the current analysis, either the AIC or BIC values show differences in the three methods.

```
AIC(Beta.ML, Beta.BC, Beta.BR)
```

```
##         df       AIC
## Beta.ML 27 -2743.354
## Beta.BC 27 -2743.254
## Beta.BR 27 -2743.254
```

```
BIC(Beta.ML, Beta.BC, Beta.BR)
```

```
##         df       BIC
## Beta.ML 27 -2570.331
## Beta.BC 27 -2570.231
## Beta.BR 27 -2570.230
```

Here, the BC and BR models haven’t shown any difference from estimation of ML, and so studying BC and BR models has no benefit.
