# Supplementary material for: Modeling determinants of women’s decision-making autonomy on agriculture and household matters in rural Ethiopia: a mixed effects beta regression tree analysis
Source: PLoS One. 2026 Sep 10;21(9):e0355932. doi: 10.1371/journal.pone.0355932 (PMC13561345; doi:10.1371/journal.pone.0355932)
Supplement: Supplementary file 3 [file pone.0355932.s001.zip › Supplementaries/S4_File.pdf]

Beta regression tree model and refitted beta regression models for each segment/node formed by the beta regression tree model

```
set.seed(1071)
n<-nrow(wdma2)
wdma2$x1<-rnorm(n)
wdma2$x2<-runif(n)
wdma2$x3<-factor(sample(0:1, n,replace = TRUE))
library(Matrix)
library(lme4)
library(betareg)
library(plotrix)
library(lmtest)
The next model is the mother model that formed the
partitions of the data.
```

```
beta_treemod5<-betatree(trans_wdma~FS+WE+PE+Age+CN+RW+WQ+SP|woreda+SR|MS+GH+x
1+x2+x3, data = wdma2,minsize=80, control = betareg.control())
summary(beta_treemod5)

## $`3`
##
## Call:
## betatree(formula = trans_wdma ~ FS + WE + PE + Age + CN + RW + WQ + SP |
##     woreda + SR, data = wdma2, control = betareg.control())
##
## Quantile residuals:
##     Min      1Q  Median      3Q     Max
## -7.1462 -0.2220  0.0617  0.2883  7.1402
##
## Coefficients (mean model with logit link):
##              Estimate Std. Error z value Pr(>|z|)
## (Intercept) -0.835855   0.103368  -8.086 6.16e-16 ***
## FS          -0.014536   0.007494  -1.940  0.05242 .
## WE           0.011006   0.007342   1.499  0.13384
## PE           0.002721   0.005731   0.475  0.63492
## Age          0.003541   0.001529   2.316  0.02055 *
## CN           0.039000   0.004418   8.827  < 2e-16 ***
## RW2         -0.190046   0.037879  -5.017 5.24e-07 ***
## RW3          0.006623   0.046179   0.143  0.88595
## WQ2          0.045511   0.046763   0.973  0.33044
## WQ3         -0.001452   0.046870  -0.031  0.97529
```

```
## WQ4         -0.015774   0.045808  -0.344  0.73057
## WQ5         -0.154283   0.047285  -3.263  0.00110 **
## SP1          0.120281   0.042435   2.835  0.00459 **
##
## Phi coefficients (precision model with log link):
##             Estimate Std. Error z value Pr(>|z|)
## (Intercept)  1.07559    0.07130  15.084  < 2e-16 ***
## woreda2     -0.14766    0.08679  -1.701 0.088882 .
## woreda3     -0.34524    0.09125  -3.783 0.000155 ***
## woreda4     -1.01825    0.08342 -12.206  < 2e-16 ***
## woreda5     -0.50130    0.09806  -5.112 3.18e-07 ***
## woreda6      0.27603    0.09773   2.824 0.004739 **
## woreda7      0.45105    0.09183   4.912 9.01e-07 ***
## woreda8      0.26437    0.07644   3.459 0.000543 ***
## woreda9     -0.25053    0.08547  -2.931 0.003378 **
## SR1          0.43012    0.04495   9.569  < 2e-16 ***
## ---
## Signif. codes:  0 '***' 0.001 '**' 0.01 '*' 0.05 '.' 0.1 ' ' 1
##
## Type of estimator: ML (maximum likelihood)
## Log-likelihood: 672.7 on 23 Df
## Pseudo R-squared: 0.0304
## Number of iterations: 44 (BFGS) + 3 (Fisher scoring)
##
## $`4`
##
## Call:
## betatree(formula = trans_wdma ~ FS + WE + PE + Age + CN + RW + WQ + SP |
##     woreda + SR, data = wdma2, control = betareg.control())
##
## Quantile residuals:
##     Min      1Q  Median      3Q     Max
## -2.4141 -0.6725  0.0249  0.5949  3.1314
##
## Coefficients (mean model with logit link):
##              Estimate Std. Error z value Pr(>|z|)
## (Intercept) -0.448086   0.606072  -0.739 0.459707
## FS           0.132227   0.060543   2.184 0.028961 *
## WE          -0.055601   0.031119  -1.787 0.073981 .
## PE           0.051219   0.029265   1.750 0.080083 .
## Age         -0.011207   0.004963  -2.258 0.023953 *
## CN           0.111389   0.035787   3.113 0.001855 **
## RW2         -0.441547   0.280899  -1.572 0.115972
## RW3         -0.285157   0.328727  -0.867 0.385691
## WQ2         -1.736416   0.360287  -4.820 1.44e-06 ***
## WQ3         -1.342788   0.348671  -3.851 0.000118 ***
## WQ4         -1.587071   0.356983  -4.446 8.76e-06 ***
## WQ5         -1.133438   0.335802  -3.375 0.000737 ***
## SP1         -0.329695   0.188807  -1.746 0.080775 .
##
```

```
## Phi coefficients (precision model with log link):
##              Estimate Std. Error z value Pr(>|z|)
## (Intercept)  0.06519    0.29312   0.222   0.8240
## woreda2      0.81809    0.32349   2.529   0.0114 *
## woreda3     -0.53316    0.37126  -1.436   0.1510
## woreda4     -1.39136    1.05857  -1.314   0.1887
## woreda5     -0.11437    0.40740  -0.281   0.7789
## woreda6     -0.23270    0.50003  -0.465   0.6417
## woreda7      0.11668    0.39964   0.292   0.7703
## woreda8      4.87379    0.62581   7.788 6.81e-15 ***
## woreda9     -0.28363    0.34778  -0.816   0.4148
## SR1         -0.22767    0.21588  -1.055   0.2916
## ---
## Signif. codes:  0 '***' 0.001 '**' 0.01 '*' 0.05 '.' 0.1 ' ' 1
##
## Type of estimator: ML (maximum likelihood)
## Log-likelihood: 111.4 on 23 Df
## Pseudo R-squared: 0.1676
## Number of iterations: 39 (BFGS) + 4 (Fisher scoring)
##
## $`5`
##
## Call:
## betatree(formula = trans_wdma ~ FS + WE + PE + Age + CN + RW + WQ + SP |
##     woreda + SR, data = wdma2, control = betareg.control())
##
## Quantile residuals:
##     Min      1Q  Median      3Q     Max
## -3.1432 -0.6815  0.4376  0.8411  1.6303
##
## Coefficients (mean model with logit link):
##               Estimate Std. Error z value Pr(>|z|)
## (Intercept)  1.7625836  0.4869364   3.620 0.000295 ***
## FS           0.0002989  0.0388995   0.008 0.993869
## WE           0.0039270  0.0196226   0.200 0.841382
## PE          -0.0189607  0.0371352  -0.511 0.609642
## Age         -0.0142996  0.0059530  -2.402 0.016302 *
## CN          -0.0154308  0.0198757  -0.776 0.437532
## RW2          0.4003865  0.1646518   2.432 0.015027 *
## RW3          0.2682568  0.2467224   1.087 0.276912
## WQ2          0.1666467  0.1613587   1.033 0.301711
## WQ3         -0.0989856  0.1994980  -0.496 0.619772
## WQ4         -0.2799149  0.2309364  -1.212 0.225479
## WQ5         -0.0019324  0.2184300  -0.009 0.992941
## SP1          0.2291803  0.1721966   1.331 0.183214
##
## Phi coefficients (precision model with log link):
##              Estimate Std. Error z value Pr(>|z|)
## (Intercept) -0.389344   0.240179  -1.621   0.1050
## woreda2     -0.449383   0.262500  -1.712   0.0869 .
```

```
## woreda3     -0.002919   0.249191  -0.012   0.9907
## woreda4     -0.152822   0.271373  -0.563   0.5733
## woreda5     -0.439463   0.291287  -1.509   0.1314
## woreda6     -0.249417   0.296424  -0.841   0.4001
## woreda7      0.251276   0.282104   0.891   0.3731
## woreda8     -0.174547   0.267080  -0.654   0.5134
## woreda9      0.014961   0.297652   0.050   0.9599
## SR1          0.180722   0.114527   1.578   0.1146
## ---
## Signif. codes:  0 '***' 0.001 '**' 0.01 '*' 0.05 '.' 0.1 ' ' 1
##
## Type of estimator: ML (maximum likelihood)
## Log-likelihood:  1172 on 23 Df
## Pseudo R-squared: 0.06037
## Number of iterations: 45 (BFGS) + 3 (Fisher scoring)
```

The above output from the BRTM, on which modeling by beta regression was done on the three segments formed by the BRTM.

Fitting Beta regression by the 'betareg' package and function for subsets of the result of BRTM (beta_treemod5) by the Bias Reduction (BR) method. Calculation of average marginal effects (AMES) followed from this model.

Three formed segments of the data by the BRTM

```
node5 <- wdma2$GH==1
node3<-wdma2$GH==0 & wdma2$MS==1
node4<-wdma2$GH==0 & wdma2$MS==2|wdma2$MS==3
```

Separate Beta Regression for the 1st segment (3rd Node of BRTM)

```
BR_Node3 <- betareg(trans_wdma~Age+CN+FS+PE+RW+SP+WE+WQ|woreda+SR, data = wdm
a2,subset=node3,type = "BR")
summary(BR_Node3)

##
## Call:
## betareg(formula = trans_wdma ~ Age + CN + FS + PE + RW + SP + WE + WQ |
##     woreda + SR, data = wdma2, subset = node3, type = "BR")
##
## Quantile residuals:
##     Min      1Q  Median      3Q     Max
## -7.1185 -0.2212  0.0617  0.2878  7.1128
##
## Coefficients (mean model with logit link):
##              Estimate Std. Error z value Pr(>|z|)
## (Intercept) -0.835768   0.103595  -8.068 7.17e-16 ***
## Age          0.003547   0.001532   2.315  0.02062 *
## CN           0.038998   0.004428   8.808  < 2e-16 ***
## FS          -0.014560   0.007510  -1.939  0.05254 .
```

```
## PE           0.002718   0.005744   0.473  0.63605
## RW2         -0.189908   0.037959  -5.003 5.65e-07 ***
## RW3          0.006619   0.046285   0.143  0.88628
## SP1          0.120313   0.042534   2.829  0.00467 **
## WE           0.011002   0.007358   1.495  0.13485
## WQ2          0.045357   0.046870   0.968  0.33318
## WQ3         -0.001556   0.046974  -0.033  0.97358
## WQ4         -0.015840   0.045911  -0.345  0.73009
## WQ5         -0.154210   0.047390  -3.254  0.00114 **
##
## Phi coefficients (precision model with log link):
##             Estimate Std. Error z value Pr(>|z|)
## (Intercept)  1.06866    0.07126  14.996  < 2e-16 ***
## woreda2     -0.14552    0.08675  -1.678 0.093437 .
## woreda3     -0.34374    0.09120  -3.769 0.000164 ***
## woreda4     -1.01519    0.08338 -12.176  < 2e-16 ***
## woreda5     -0.50063    0.09799  -5.109 3.24e-07 ***
## woreda6      0.27548    0.09768   2.820 0.004798 **
## woreda7      0.45040    0.09178   4.908 9.22e-07 ***
## woreda8      0.26635    0.07640   3.486 0.000490 ***
## woreda9     -0.24940    0.08542  -2.920 0.003506 **
## SR1          0.43015    0.04493   9.575  < 2e-16 ***
## ---
## Signif. codes:  0 '***' 0.001 '**' 0.01 '*' 0.05 '.' 0.1 ' ' 1
##
## Type of estimator: BR (bias-reduced)
## Log-likelihood: 672.7 on 23 Df
## Pseudo R-squared: 0.0304
## Number of iterations: 44 (BFGS) + 13 (quasi Fisher scoring)
```

Separate Beta Regression for the 2nd segment (4th Node of BRTM)

```
BR_Node4<-betareg(trans_wdma~Age+CN+FS+PE+RW+SP+WE+WQ|woreda+SR, data = wdma2
,subset=node4,type = "BR")
summary(BR_Node4)
```

```
##
## Call:
## betareg(formula = trans_wdma ~ Age + CN + FS + PE + RW + SP + WE + WQ |
##     woreda + SR, data = wdma2, subset = node4, type = "BR")
##
## Quantile residuals:
##     Min      1Q  Median      3Q     Max
## -2.4355 -0.3955 -0.0443  0.3863  2.4866
##
## Coefficients (mean model with logit link):
##              Estimate Std. Error z value Pr(>|z|)
## (Intercept)  0.059135   0.732208   0.081 0.935631
## Age          0.010369   0.007660   1.354 0.175866
## CN           0.012915   0.039512   0.327 0.743777
```

```
## FS           0.096079   0.064537   1.489 0.136555    
## PE          -0.013122   0.032237  -0.407 0.683970    
## RW2          0.009189   0.266240   0.035 0.972468    
## RW3         -0.119891   0.359257  -0.334 0.738592    
## SP1          0.123747   0.302250   0.409 0.682232    
## WE          -0.030051   0.034888  -0.861 0.389044    
## WQ2         -1.167399   0.319933  -3.649 0.000263 ***
## WQ3         -1.239650   0.356356  -3.479 0.000504 ***
## WQ4         -1.165277   0.352708  -3.304 0.000954 ***
## WQ5         -1.229777   0.363492  -3.383 0.000716 ***
## 
## Phi coefficients (precision model with log link):
##             Estimate Std. Error z value Pr(>|z|)  
## (Intercept) -0.37631    0.27199  -1.384   0.1665  
## woreda2      0.22849    0.29897   0.764   0.4447  
## woreda3     -0.30738    0.35400  -0.868   0.3852  
## woreda4     -1.07062    0.51271  -2.088   0.0368 *
## woreda5      0.51419    0.37402   1.375   0.1692  
## woreda6     -0.12813    0.49545  -0.259   0.7959  
## woreda7      0.21709    0.33587   0.646   0.5181  
## woreda8     -0.30960    0.42232  -0.733   0.4635  
## woreda9     -0.37638    0.32236  -1.168   0.2430  
## SR1         -0.06259    0.18366  -0.341   0.7333  
## ---
## Signif. codes:  0 '***' 0.001 '**' 0.01 '*' 0.05 '.' 0.1 ' ' 1 
## 
## Type of estimator: BR (bias-reduced)
## Log-likelihood: 174.3 on 23 Df
## Pseudo R-squared: 0.1151
## Number of iterations: 34 (BFGS) + 29 (quasi Fisher scoring)
```

Separate Beta Regression for the 3rd segment (5th Node of BRTM)

```
BR_Node5 <- betareg(trans_wdma~Age+CN+FS+PE+RW+SP+WE+WQ|woreda+SR, data = wdm
a2,subset=node5,type = "BR")
summary(BR_Node5)

## 
## Call:
## betareg(formula = trans_wdma ~ Age + CN + FS + PE + RW + SP + WE + WQ | 
##     woreda + SR, data = wdma2, subset = node5, type = "BR")
## 
## Quantile residuals:
##     Min      1Q  Median      3Q     Max 
## -3.0113 -0.6891  0.4346  0.7890  1.6502 
## 
## Coefficients (mean model with logit link):
##              Estimate Std. Error z value Pr(>|z|)    
## (Intercept)  1.810208   0.452820   3.998  6.4e-05 ***
## Age         -0.014080   0.005549  -2.537   0.0112 *  
```

```
## CN          -0.004461   0.018653  -0.239   0.8110
## FS          -0.014677   0.036698  -0.400   0.6892
## PE          -0.014314   0.036271  -0.395   0.6931
## RW2          0.344690   0.154485   2.231   0.0257 *
## RW3          0.190794   0.235331   0.811   0.4175
## SP1          0.164329   0.156040   1.053   0.2923
## WE          -0.009773   0.018417  -0.531   0.5957
## WQ2          0.164458   0.151028   1.089   0.2762
## WQ3         -0.089821   0.193882  -0.463   0.6432
## WQ4         -0.287776   0.224045  -1.284   0.1990
## WQ5         -0.009347   0.214684  -0.044   0.9653
##
## Phi coefficients (precision model with log link):
##             Estimate Std. Error z value Pr(>|z|)
## (Intercept) -0.44403    0.23166  -1.917   0.0553 .
## woreda2     -0.42446    0.25283  -1.679   0.0932 .
## woreda3     -0.01835    0.23755  -0.077   0.9384
## woreda4     -0.18007    0.26338  -0.684   0.4942
## woreda5     -0.50247    0.27563  -1.823   0.0683 .
## woreda6     -0.22363    0.26435  -0.846   0.3976
## woreda7      0.42007    0.26496   1.585   0.1129
## woreda8     -0.13029    0.25921  -0.503   0.6152
## woreda9     -0.03196    0.29116  -0.110   0.9126
## SR1          0.20837    0.11155   1.868   0.0618 .
## ---
## Signif. codes:  0 '***' 0.001 '**' 0.01 '*' 0.05 '.' 0.1 ' ' 1
##
## Type of estimator: BR (bias-reduced)
## Log-likelihood:  1328 on 23 Df
## Pseudo R-squared: 0.05401
## Number of iterations: 47 (BFGS) + 15 (quasi Fisher scoring)
```

These separate beta regression models were employed to estimate average marginal effects (AMEs).
