# Supplementary material for: Modeling determinants of women’s decision-making autonomy on agriculture and household matters in rural Ethiopia: a mixed effects beta regression tree analysis
Source: PLoS One. 2026 Sep 10;21(9):e0355932. doi: 10.1371/journal.pone.0355932 (PMC13561345; doi:10.1371/journal.pone.0355932)
Supplement: Peer review history

# Peer review: Decision Letter 0

**DOI:** 10.1371/journal.pone.0355932.r001

**Authors:** Ayodeji Oginni

8 May 2026

Dear Dr. Dadi,

Thank you for submitting your manuscript to PLOS ONE. After careful consideration, we feel that it has merit but does not fully meet PLOS ONE’s publication criteria as it currently stands. Therefore, we invite you to submit a revised version of the manuscript that addresses the points raised during the review process.

Please submit your revised manuscript by Jun 22 2026 11:59PM. If you will need more time than this to complete your revisions, please reply to this message or contact the journal office at plosone@plos.org. When you're ready to submit your revision, log on to https://www.editorialmanager.com/pone/ and select the 'Submissions Needing Revision' folder to locate your manuscript file.

- A letter that responds to each point raised by the academic editor and reviewer(s). You should upload this letter as a separate file labeled 'Response to Reviewers'.
- A marked-up copy of your manuscript that highlights changes made to the original version. You should upload this as a separate file labeled 'Revised Manuscript with Track Changes'.
- An unmarked version of your revised paper without tracked changes. You should upload this as a separate file labeled 'Manuscript'.

If you would like to make changes to your financial disclosure, please include your updated statement in your cover letter. Guidelines for resubmitting your figure files are available below the reviewer comments at the end of this letter.

If applicable, we recommend that you deposit your laboratory protocols in protocols.io to enhance the reproducibility of your results. Protocols.io assigns your protocol its own identifier (DOI) so that it can be cited independently in the future. For instructions see: https://journals.plos.org/plosone/s/submission-guidelines#loc-laboratory-protocols. Additionally, PLOS ONE offers an option for publishing peer-reviewed Lab Protocol articles, which describe protocols hosted on protocols.io. Read more information on sharing protocols at https://plos.org/protocols?utm_medium=editorial-email&utm_source=authorletters&utm_campaign=protocols.

As the corresponding author, your ORCID iD is verified in the submission system and will appear in the published article. PLOS supports the use of ORCID, and we encourage all coauthors to register for an ORCID iD and use it as well. Please encourage your coauthors to verify their ORCID iD within the submission system before final acceptance, as unverified ORCID iDs will not appear in the published article. *Only*  the individual author can complete the verification step; PLOS staff *cannot*  verify ORCID iDs on behalf of authors.

We look forward to receiving your revised manuscript.

Kind regards,

Ayodeji Babatunde Oginni

Academic Editor

PLOS One

Journal Requirements:

When submitting your revision, we need you to address these additional requirements.

1. Please ensure that your manuscript meets PLOS ONE's style requirements, including those for file naming. The PLOS ONE style templates can be found at

https://journals.plos.org/plosone/s/file?id=wjVg/PLOSOne_formatting_sample_main_body.pdf and

https://journals.plos.org/plosone/s/file?id=ba62/PLOSOne_formatting_sample_title_authors_affiliations.pdf

2. We note that you have indicated that there are restrictions to data sharing for this study. PLOS only allows data to be available upon request if there are legal or ethical restrictions on sharing data publicly. For more information on unacceptable data access restrictions, please see http://journals.plos.org/plosone/s/data-availability#loc-unacceptable-data-access-restrictions.

Before we proceed with your manuscript, please address the following prompts:

a) If there are ethical or legal restrictions on sharing a de-identified data set, please explain them in detail (e.g., data contain potentially identifying or sensitive patient information, data are owned by a third-party organization, etc.) and who has imposed them (e.g., a Research Ethics Committee or Institutional Review Board, etc.). Please also provide contact information for a data access committee, ethics committee, or other institutional body to which data requests may be sent.

b) If there are no restrictions, please upload the minimal anonymized data set necessary to replicate your study findings to a stable, public repository and provide us with the relevant URLs, DOIs, or accession numbers. For a list of recommended repositories, please see

https://journals.plos.org/plosone/s/recommended-repositories. You also have the option of uploading the data as Supporting Information files, but we would recommend depositing data directly to a data repository if possible.

We will update your Data Availability statement on your behalf to reflect the information you provide.

3. If the reviewer comments include a recommendation to cite specific previously published works, please review and evaluate these publications to determine whether they are relevant and should be cited. There is no requirement to cite these works unless the editor has indicated otherwise.

4. Please review your reference list to ensure that it is complete and correct. If you have cited papers that have been retracted, please include the rationale for doing so in the manuscript text, or remove these references and replace them with relevant current references. Any changes to the reference list should be mentioned in the rebuttal letter that accompanies your revised manuscript. If you need to cite a retracted article, indicate the article’s retracted status in the References list and also include a citation and full reference for the retraction notice.

[Note: HTML markup is below. Please do not edit.]

Reviewers' comments:

Reviewer's Responses to Questions

**Comments to the Author**

1. Is the manuscript technically sound, and do the data support the conclusions?

Reviewer #1: Yes

Reviewer #2: Yes

Reviewer #3: Yes

Reviewer #4: Partly

Reviewer #5: Yes

**********

2. Has the statistical analysis been performed appropriately and rigorously?

Reviewer #1: Yes

Reviewer #2: Yes

Reviewer #3: Yes

Reviewer #4: Yes

Reviewer #5: Yes

**********

3. Have the authors made all data underlying the findings in their manuscript fully available?

The PLOS Data policy

Reviewer #1: Yes

Reviewer #2: Yes

Reviewer #3: Yes

Reviewer #4: Yes

Reviewer #5: No

**********

4. Is the manuscript presented in an intelligible fashion and written in standard English?

Reviewer #1: Yes

Reviewer #2: Yes

Reviewer #3: Yes

Reviewer #4: Yes

Reviewer #5: Yes

**********

Reviewer #1: General Comments

This manuscript uses a mixed-effects beta regression tree approach to investigate the determinants of women’s decision-making autonomy in rural Ethiopian agriculture and households. The topic is timely and relevant, the methodology appropriate and innovative, and the analysis technically sound. The manuscript is well-organized, clearly written, and the conclusions are data-supported. I recommend minor revisions to improve clarity, interpretation, and presentation.

Major Comments

1- Methodological clarity: The description of the statistical model would benefit from additional clarification regarding assumptions, parameter tuning, and validation procedures. A brief justification for selecting this approach over alternative models would improve transparency.

2- Interpretation of results: The discussion could be strengthened by deeper interpretation of the key predictors and their socio-economic implications, with clearer linkage to existing literature.

3- Policy implications: The policy recommendations are appropriate but could be expanded with more concrete and actionable suggestions.

Minor Comments :

1- Minor grammatical and typographical errors should be corrected, such as occasional subject–verb agreement issues, inconsistent use of articles (e.g., “a”, “an”, “the”), and minor punctuation errors, mainly in the Introduction and Discussion sections.

2- F igures and tables would benefit from more detailed captions. For example, in Table X and Figure Y, abbreviations such as “EA”, “HH”, and “DMI” should be clearly defined, and a brief explanation of key variables and units would improve clarity.

3- Organization of the introduction could be slightly improved. For instance, merging the background discussion on women’s empowerment with agricultural decision-making may reduce repetition and enhance logical flow.

Recommendation

Overall, this is a technically sound and valuable study. I recommend minor revision before acceptance.

Reviewer #2: The work is good and can be published after addressing the minor comments

1. Revise the abstract mentioning the main work and main outcomes

2. Look the whole manuscript regarding the spelling and grammatical errors and fix them

3. At the end of the introduction write the novelty of the current work clearly

4. Cite the references properly where from the authors got the idea

5. What are the limitations and future recommendations of the current work in the end of the conclusion section.

Reviewer #3: The authors made a meaningful methodological contribution by applying a mixed-effects beta regression tree to analyze WDMA in rural Ethiopia. I suggest the followings to improve this study:

- Report intra-class correlation (ICC).

- Provide reliability of data by apply any suitable test e.g., Cronbach’s alpha test

- Provide cross-validation for tree model.

- Distinguish agricultural vs household autonomy if conceptually separable.

Reviewer #4: Dear Authors,

I have carefully read your manuscript on women’s decision-making autonomy (WDMA) in rural Ethiopia. The topic is very relevant for sociology and development studies. Using a "Mixed Effects Beta Regression Tree" is a strong point of this paper, as it captures the complexity of household dynamics better than simpler models. However, I have several concerns that need to be addressed to improve the quality and clarity of the paper.

Major Points:

1. Definitions in the Introduction:

The paper often uses "autonomy" and "empowerment" interchangeably. In sociological literature (e.g., Naila Kabeer, 1999), these concepts are distinct. I suggest the authors provide a clearer definition of "autonomy" in their specific context. Does it mean the woman has the "final say," or is she simply "consulted"? This distinction is crucial for interpreting your results.

2. Local Context:

Ethiopia has diverse cultural and land-tenure systems, especially in Oromia and the Southern regions. These local norms often influence women more than age or wealth. I think the paper needs a small paragraph to explain if these regional and ethnic differences were considered in the sociological analysis.

3. Measuring Participation (Methodology):

The study considers a woman as "participating" if "any family member" is in a social group. I find this to be a weakness. If only the husband is part of a group, it might actually increase his power over the wife rather than increasing her agency. It would be better to focus on the "woman’s own participation."

4. Potential Bias in Interviews:

The manuscript does not mention if the interviews were conducted in private. In rural villages, the presence of a husband or male relatives during the interview can significantly bias a woman’s answers about her decision-making power. This should be mentioned as a limitation.

5. Contradiction in Religion Results (Table 3 vs. Abstract):

There is a serious discrepancy between the Abstract and the Results. In the Abstract, the authors state that being Orthodox (compared to Muslim) is associated with higher autonomy. However, in Table 3 (Node 3), which covers the majority of the sample (n=3961), the coefficient for Orthodox is significantly negative (-0.0468). This indicates lower autonomy for married women in male-led households. Please correct this in the text and discuss why the effect of religion is negative in Node 3 but positive in Node 5.

6. Discussion on Wealth Paradox:

The finding that being in the "Richest" quintile reduces autonomy (-0.0380 in Node 3) is very interesting. Usually, more wealth is expected to increase power, but your data shows the opposite. I suggest discussing this "prestige" factor—do wealthy rural families keep women away from decisions to maintain social status?

Conclusion:

While the statistical work is solid, the paper needs a deeper sociological discussion to explain these patterns. The contradiction regarding the religion variable must be addressed. I recommend a Major Revision.

________________________________________

Reviewer #5: The manuscript addresses an important and relevant topic concerning women’s decision-making autonomy in agricultural and household contexts in rural Ethiopia. The use of a relatively large dataset and the attempt to capture heterogeneity through a mixed-effects beta regression tree model are notable strengths of the study.

However, several issues should be addressed to improve the quality and clarity of the manuscript:

Statistical clarity and justification:

While the use of the beta regression tree model is innovative and appropriate for bounded outcomes and heterogeneous data, the manuscript lacks sufficient justification for selecting this model over alternative approaches. The discussion of model selection (based primarily on AIC) should be expanded, and more information on model diagnostics and goodness-of-fit should be provided.

Interpretation of findings:

Some findings appear counterintuitive, particularly the negative association between household wealth and women’s decision-making autonomy in certain subgroups. These results require deeper theoretical engagement and clearer contextual explanation. Strengthening the linkage between empirical findings and existing literature would enhance the contribution of the study.

Data availability and transparency:

The data underlying the findings are not fully publicly available and require access through a third party. While ethical constraints may justify this, the data availability statement should provide clearer details on the procedure for requesting access and the conditions under which the data can be obtained. This would improve transparency and alignment with journal policies.

Organization and presentation:

Some sections—particularly the methods and discussion—are overly detailed and could benefit from improved structure and conciseness. Reducing redundancy and focusing on key analytical steps would improve readability.

Language and readability:

The manuscript is generally understandable; however, it would benefit from language editing to improve clarity, reduce repetition, and refine sentence structure. Several sections contain long and complex sentences that may hinder comprehension.

Overall, the manuscript has merit and contributes to the literature on women’s empowerment and decision-making autonomy. With revisions addressing the points above, the paper could be strengthened significantly in terms of clarity, rigor, and interpretability.

**********

what does this mean?). If published, this will include your full peer review and any attached files.

If you choose “no”, your identity will remain anonymous but your review may still be made public.

**Do you want your identity to be public for this peer review?**  For information about this choice, including consent withdrawal, please see our Privacy Policy

Reviewer #1: No

Reviewer #2: No

Reviewer #3: No

Reviewer #4: No

Reviewer #5: No

**********

[NOTE: If reviewer comments were submitted as an attachment file, they will be attached to this email and accessible via the submission site. Please log into your account, locate the manuscript record, and check for the action link "View Attachments". If this link does not appear, there are no attachment files.]

To ensure your figures meet our technical requirements, please review our figure guidelines: https://journals.plos.org/plosone/s/figures

You may also use PLOS’s free figure tool, NAAS, to help you prepare publication quality figures: https://journals.plos.org/plosone/s/figures#loc-tools-for-figure-preparation.

NAAS will assess whether your figures meet our technical requirements by comparing each figure against our figure specifications.

Attachment Submitted filename: Reviewer Report.docx

# Author comment: Author response to Decision Letter 1

**DOI:** 10.1371/journal.pone.0355932.r002

20 Jul 2026

Rebuttal letter

PONE-D-25-54211

Modeling determinants of women’s decision-making autonomy on agriculture and household matters in rural Ethiopia: a mixed effects beta regression tree analysis

Dear Editor and referee,

Thank you very much for giving us the opportunity to revise our manuscript. We appreciate all comments and suggestions, which we believe have helped improve the quality of the manuscript. Our responses to the editor’s and reviewers' comments are provided below in a point-by-point format.

Best regards,

Teshome K. Dadi & Co-authors

Reviewers’ Comment

Reviewer #1:

General Comments

This manuscript uses a mixed-effects beta regression tree approach to investigate the determinants of women’s decision-making autonomy in rural Ethiopian agriculture and households. The topic is timely and relevant, the methodology appropriate and innovative, and the analysis technically sound. The manuscript is well-organized, clearly written, and the conclusions are data-supported. I recommend minor revisions to improve clarity, interpretation, and presentation.

Response: Thank you very much for your positive and encouraging feedback. We greatly appreciate your recommendation for minor revisions and we have now carefully addressed all comments.

Major Comments

1- Methodological clarity: The description of the statistical model would benefit from additional clarification regarding assumptions, parameter tuning, and validation procedures. A brief justification for selecting this approach over alternative models would improve transparency.

Response: We thank the reviewer for this valuable suggestion. A detailed description of the statistical model, including the underlying theory, mathematical formulas, model assumptions, and selection rationale, was already provided in the “Statistical Analysis” subsection (pages 6–8).

To further improve clarity and transparency as requested, we have added a concise summary of the model and its key assumptions in the body of the manuscript in track change with Latex in black blue color.

2- Interpretation of results: The discussion could be strengthened by deeper interpretation of the key predictors and their socio-economic implications, with clearer linkage to existing literature.

Response: We believe that our discussion already provides these interpretations and linkage to literature. We believe the discussion is already long and detailed, but we have made some revisions in response to this comment (p. 18).

3- Policy implications: The policy recommendations are appropriate but could be expanded with more concrete and actionable suggestions.

Response: We have now expanded the policy recommendations (p.22)

Minor Comments:

1- Minor grammatical and typographical errors should be corrected, such as occasional subject–verb agreement issues, inconsistent use of articles (e.g., “a”, “an”, “the”), and minor punctuation errors, mainly in the Introduction and Discussion sections.

Response: Thank you. We have reviewed and addressed grammatical and typographical errors in the manuscript.

2- Figures and tables would benefit from more detailed captions. For example, in Table X and Figure Y, abbreviations such as “EA”, “HH”, and “DMI” should be clearly defined, and a brief explanation of key variables and units would improve clarity.

Response: We have defined variables with their measurement under the ‘Measurements and operational definitions’ sub section (page 5 to 6). In all the figures and tables also, abbreviations have now been clearly defined.

3- Organization of the introduction could be slightly improved. For instance, merging the background discussion on women’s empowerment with agricultural decision-making may reduce repetition and enhance logical flow.

Response: We have made some edits in the background section to improve clarity and flow.

Recommendation

Overall, this is a technically sound and valuable study. I recommend minor revision before acceptance.

Response: We sincerely thank the reviewer for the positive evaluation of our manuscript and for recognizing its technical soundness and scientific value. We greatly appreciate your constructive feedback and recommendation for minor revision.

Reviewer #2:

The work is good and can be published after addressing the minor comments.

Response: We sincerely thank the reviewer for the positive assessment of our work and for recommending its publication.

1. Revise the abstract mentioning the main work and main outcomes.

Response: The abstract already summarizes the main methodology employed and the key factors associated with women's decision-making autonomy in heterogeneous households. We would also like to note that the journal has a strict word limit for the abstract, which limits the amount of detail that can be included.

2. Look the whole manuscript regarding the spelling and grammatical errors and fix them.

Response: We would like to thank the reviewer for this helpful suggestion. We have now checked the manuscript for spelling and grammatical error.

3. At the end of the introduction write the novelty of the current work clearly

Response: Thank you very much. We updated the introduction section by stating the novelty of our work

4. Cite the references properly where from the authors got the idea.

Response: Thank you. We have gone through the reference list and have updated citation which was incorrect.

5. What are the limitations and future recommendations of the current work in the end of the conclusion section?

Response: Thank you for this valuable suggestion. We have included limitations and provided recommendations for future studies to build upon this work.

Reviewer #3:

The authors made a meaningful methodological contribution by applying a mixed-effects beta regression tree to analyze WDMA in rural Ethiopia. I suggest the followings to improve this study:

Response: Thank you for your encouragement!

- Report intra-class correlation (ICC).

Response: We have reported the ICC under the sub section of Survey Round on page #6.

- Provide reliability of data by apply any suitable test e.g., Cronbach’s alpha test.

Response: We have calculated the Cronbach’s alpha test and reported in the revised manuscript (p. 5)

- Provide cross-validation for tree model.

Response: We thank the reviewer for raising this point. In this study, it was through parameter stability test that the robustness of the Beta Regression Tree model was evaluated. This process confirmed that the main findings regarding the determinants of women’s decision-making autonomy were stable and not overly sensitive to specific parameter choices. Although cross-validation is a valuable tool for assessing predictive performance and guarding against over fitting, our primary objective was interpretive inference on the complex relationships and interactions within the data, rather than pure predictive accuracy. Given the bounded nature of the outcome variable and the clustered survey design, we believe the parameter stability analysis provided sufficient evidence of model reliability.

- Distinguish agricultural vs household autonomy if conceptually separable.

Response: Thank you for your suggestion. They are not conceptually separable in the context of this study.

Reviewer #4:

Dear Authors,

I have carefully read your manuscript on women’s decision-making autonomy (WDMA) in rural Ethiopia. The topic is very relevant for sociology and development studies. Using a "Mixed Effects Beta Regression Tree" is a strong point of this paper, as it captures the complexity of household dynamics better than simpler models. However, I have several concerns that need to be addressed to improve the quality and clarity of the paper.

Response: We sincerely thank the reviewer for the careful and thoughtful evaluation of our manuscript and for recognizing the relevance of our study, particularly to the fields of sociology and development studies, where understanding women's decision-making autonomy is of considerable importance. We are especially grateful for the reviewer's recognition of the methodological strength of our study, particularly the application of the Mixed Effects Beta Regression Tree model to capture the complexity and heterogeneity of household decision making.

Major Points:

1. Definitions in the Introduction:

The paper often uses "autonomy" and "empowerment" interchangeably. In sociological literature (e.g., Naila Kabeer, 1999), these concepts are distinct. I suggest the authors provide a clearer definition of "autonomy" in their specific context. Does it mean the woman has the "final say," or is she simply "consulted"? This distinction is crucial for interpreting your results.

Response: Thank you for the feedback. Regarding autonomy and empowerment, we would like to clarify that we did not use the two interchangeably. We fully acknowledge that these are related but conceptually distinct constructs, and we have intentionally avoided using them interchangeably throughout the manuscript. Consistent with the literature, we recognize that women's decision-making autonomy is one of the key domains of women's empowerment, rather than a synonym for empowerment itself. We also acknowledge that some previous studies have used women's decision-making autonomy as a proxy or indicator of women's empowerment, and we have cited this literature where appropriate. Nevertheless, in our study, we have maintained a clear conceptual distinction between the two constructs and have consistently focused on women's decision-making autonomy as the outcome of interest. To further clarify this point, we have made minor edits to the wording to ensure that the distinction is clear and unambiguous to readers.

The second concern is about the meaning of autonomy. The contextual definition of autonomy and its measurements in our study was described in ‘Measurements and operational definitions’ sub section of the method section on page 5. In this study, women's decision-making autonomy refers to a woman's having the final say, either independently or jointly with her husband/partner, on household and agricultural decisions, consistent with the definition used in the survey instrument and previous studies. We have addressed this concern in the body of the manuscript on page 5.

2. Local Context:

Ethiopia has diverse cultural and land-tenure systems, especially in Oromia and the Southern regions. These local norms often influence women more than age or wealth. I think the paper needs a small paragraph to explain if these regional and ethnic differences were considered in the sociological analysis.

Response: We thank the reviewer for this valuable observation. The present study was however based on secondary data that were not originally collected to specifically investigate the sociological effects of cultural norms, ethnicity, or land-tenure systems on women's decision-making autonomy. Thus, these variables were not available for analysis and could not be explicitly examined in our models. We have acknowledged this limitation in the Limitations section of the revised manuscript and recommend that future studies incorporate detailed information for further exploration of the potential predictors that could influence women's decision-making autonomy.

3. Measuring Participation (Methodology):

The study considers a woman as "participating" if "any family member" is in a social group. I find this to be a weakness. If only the husband is part of a group, it might actually increase his power over the wife rather than increasing her agency. It would be better to focus on the "woman’s own participation."

Response: We thank the reviewer for this insightful comment. We agree that a woman's own participation in social groups is conceptually more relevant to women's decision-making autonomy than the participation of any household member. However, this study was based on secondary data, and the available variable only indicated whether any household member participated in a social group, without identifying which household member was involved. In the previous version of the manuscript, we interpreted this variable under the assumption that participation by any household member could facilitate information diffusion and potentially enhance women's decision-making autonomy. We acknowledge the reviewer's important point that, if the participant was the husband or another household member, such participation may instead reinforce existing household power dynamics and may not necessarily improve, and could even reduce a woman's autonomy. In light of this valuable observation, we have revised the manuscript to explicitly acknowledge this limitation in the Limitations sections and advise readers to interpret the findings for this variable with appropriate caution. Future studies should distinguish women's own participation from that of other household members to better assess its influence on women's decision-making autonomy.

4. Potential Bias in Interviews:

The manuscript does not mention if the interviews were conducted in private. In rural villages, the presence of a husband or male relatives during the interview can significantly bias a woman’s answers about her decision-making power. This should be mentioned as a limitation.

Response: We are thankful about the comment and we acknowledge that interview privacy is an important consideration when collecting data on sensitive topics such as women's decision-making autonomy. However, the present study was based on secondary data. The survey was conducted in accordance with established ethical standards, including obtaining informed consent and ensuring participants' confidentiality, as documented in the survey protocol. The dataset does not provide information on the interview setting (e.g., whether interviews were conducted in private), and we therefore cannot comment further on this aspect but we have no reason to suspect that the interviews were not conducted in private. We have no evidence to suggest that the quality of the data was compromised by the presence of other household members during the interviews.

5. Contradiction in Religion Results (Table 3 vs. Abstract):

There is a serious discrepancy between the Abstract and the Results. In the Abstract, the authors state that being Orthodox (compared to Muslim) is associated with higher autonomy. However, in Table 3 (Node 3), which covers the majority of the sample (n=3961), the coefficient for Orthodox is significantly negative (-0.0468). This indicates lower autonomy for married women in male-led households. Please correct this in the text and discuss why the effect of religion is negative in Node 3 but positive in Node 5.

Response: Thank you for this important picking of the typographic error we made in the abstract, which we have now corrected.

6. Discussion on Wealth Paradox:

The finding that being in the "Richest" quintile reduces autonomy (-0.0380 in Node 3) is very interesting. Usually, more wealth is expected to increase power, but your data shows the opposite. I suggest discussing this "prestige" factor—do wealthy rural families keep women away from decisions to maintain social status?

Response: Thank you for this comment, which we have now incorporated in the manuscript on page 18.

Conclusion:

While the statistical work is solid, the paper needs a deeper sociological discussion to explain these patterns. The contradiction regarding the religion variable must be addressed. I recommend a Major Revision.

Response: We thank the reviewer and we have addressed the valuable comments provided.

________________________________________

Reviewer #5:

The manuscript addresses an important and relevant topic concerning women’s decision-making autonomy in agricultural and household contexts in rural Ethiopia. The use of a relatively large dataset and the attempt to capture heterogeneity through a mixed-effects beta regression tree model are notable strengths of the study.

Response: We greatly appreciate your recognition of the importance of the research topic, the use of a large nationally representative dataset, and the application of the mixed-effects beta regression tree model to capture heterogeneity in women's decision-making autonomy. Your thoughtful feedback is greatly valued an

Attachment Submitted filename: Response to Reviewers.docx

# Editor report: Decision Letter 1

**DOI:** 10.1371/journal.pone.0355932.r003

**Authors:** Ayodeji Oginni

28 Jul 2026

Modeling determinants of women’s decision-making autonomy on agriculture and household matters in rural Ethiopia: a mixed effects beta regression tree analysis

PONE-D-25-54211R1

Dear Dr. Dadi,

We’re pleased to inform you that your manuscript has been judged scientifically suitable for publication and will be formally accepted for publication once it meets all outstanding technical requirements.

Within one week, you’ll receive an e-mail detailing the required amendments. When these have been addressed, you’ll receive a formal acceptance letter and your manuscript will be scheduled for publication.

An invoice will be generated when your article is formally accepted. Please note, if your institution has a publishing partnership with PLOS and your article meets the relevant criteria, all or part of your publication costs will be covered. Please make sure your user information is up-to-date by logging into Editorial Manager at Editorial Manager® and clicking the ‘Update My Information' link at the top of the page. For questions related to billing, please contact billing support.

If your institution or institutions have a press office, please notify them about your upcoming paper to help maximize its impact. If they’ll be preparing press materials, please inform our press team as soon as possible -- no later than 48 hours after receiving the formal acceptance. Your manuscript will remain under strict press embargo until 2 pm Eastern Time on the date of publication. For more information, please contact onepress@plos.org.

Kind regards,

Ayodeji Babatunde Oginni

Academic Editor

PLOS One

Additional Editor Comments (optional):

Reviewers' comments:
